# Trends in the Immunomodulatory Effects of *Cordyceps militaris*: Total Extracts, Polysaccharides and Cordycepin

**DOI:** 10.3389/fphar.2020.575704

**Published:** 2020-11-30

**Authors:** Chun-Ting Lee, Keng-Shiang Huang, Jei-Fu Shaw, Jung-Ren Chen, Wen-Shuo Kuo, Gangxu Shen, Alexandru Mihai Grumezescu, Alina Maria Holban, Yi-Ting Wang, Jun-Sheng Wang, Yi-Ping Hsiang, Yu-Mei Lin, Hsiao-Han Hsu, Chih-Hui Yang

**Affiliations:** ^1^ The School of Chinese Medicine for Post-Baccalaureate, I-Shou University, Kaohsiung, Taiwan; ^2^ Amulette Chinese Medicine Clinic, Tainan City, Taiwan; ^3^ Department of Biological Science and Technology, I-Shou University, Kaohsiung, Taiwan; ^4^ School of Chemistry and Materials Science, Nanjing University of Information Science and Technology, Nanjing, China; ^5^ Department of Science and Engineering of Oxide Materials and Nanomaterials, Polytechnic University of Bucharest, Bucharest, Romania; ^6^ Department of Microbiology and Immunology, University of Bucharest, Bucharest, Romania; ^7^ Taiwan Instrument Research Institute, National Applied Research Laboratories, Taipei, Taiwan; ^8^ Pharmacy Department of E-Da Hospital, Kaohsiung City, Taiwan

**Keywords:** *Cordyceps militaris*, immunomodulation, polysaccharides, cordycepin, type 1 immunity, type 2 immunity

## Abstract

*Cordyceps militaris* (*C. militaris*) is a fungus with a long history of widespread use in folk medicine, and its biological and medicinal functions are well studied. A crucial pharmacological effect of *C. militaris* is immunomodulation. In this review, we catalog the immunomodulatory effects of different extracts of *C. militaris*, namely total extracts, polysaccharides and cordycepin*.* Total extracts obtained using water or 50% ethyl alcohol and polysaccharides from *C. militaris* were discovered to tend to promote type 1 immunity, whereas total extracts obtained using 70–80% ethyl alcohol and cordycepin from *C. militaris* were more likely to promote type 2 immunity. This article is the first to classify the immunomodulatory effects of different extracts of *C. militaris*. In addition, we discovered a relationship between different segments or extracts and differing types of immunity. This review can provide the readers a comprehensive understanding on the immunomodulatory effects of the precious folk medicine and guidance on its use for both health people and those with an immunodeficiency.

## Introduction

**FIGURE 1:**
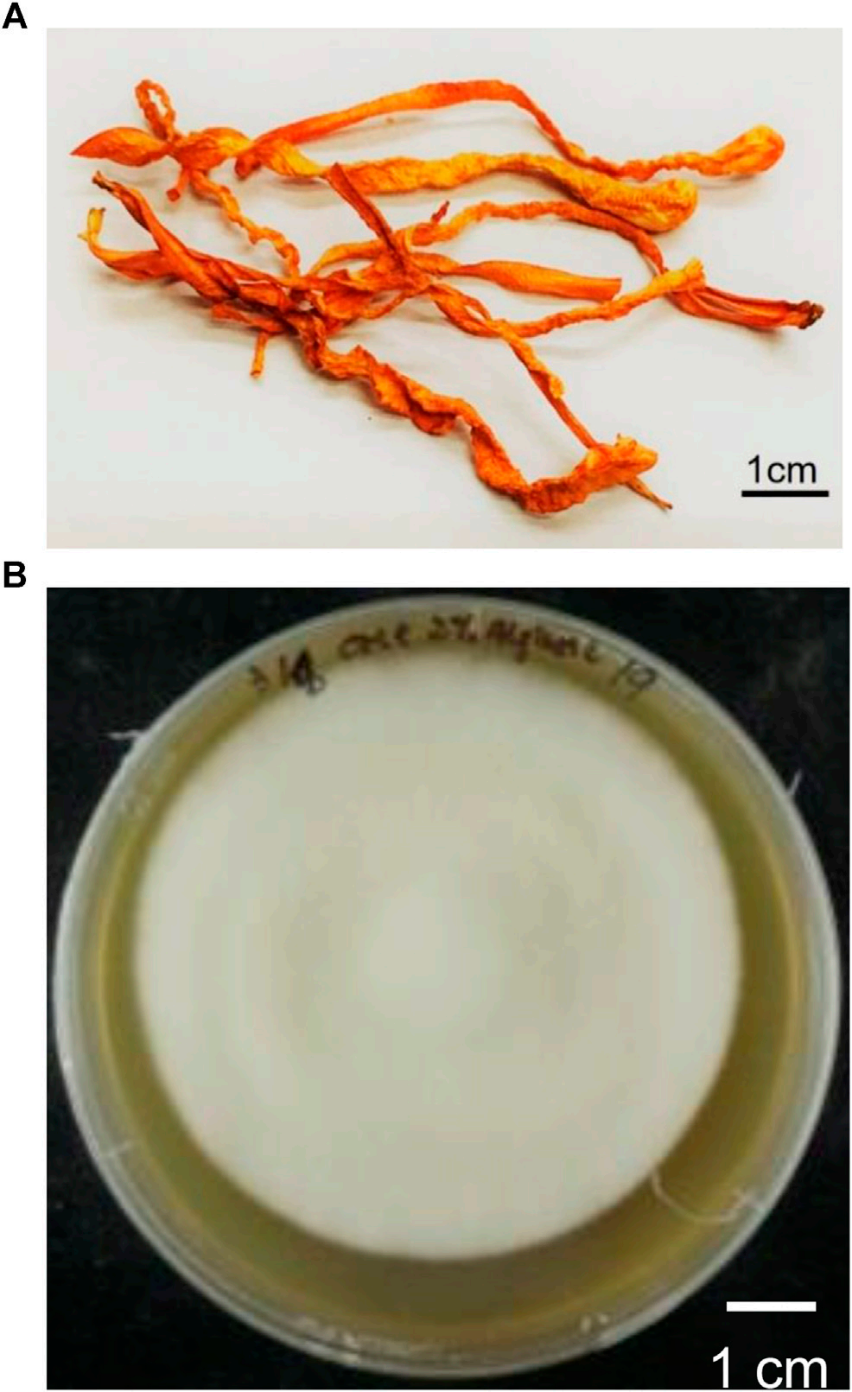
**(A)** The dried fruiting body products of *C. militaris* **(B)** The cultured mycelia of *C. militaris*.

**FIGURE 2:**
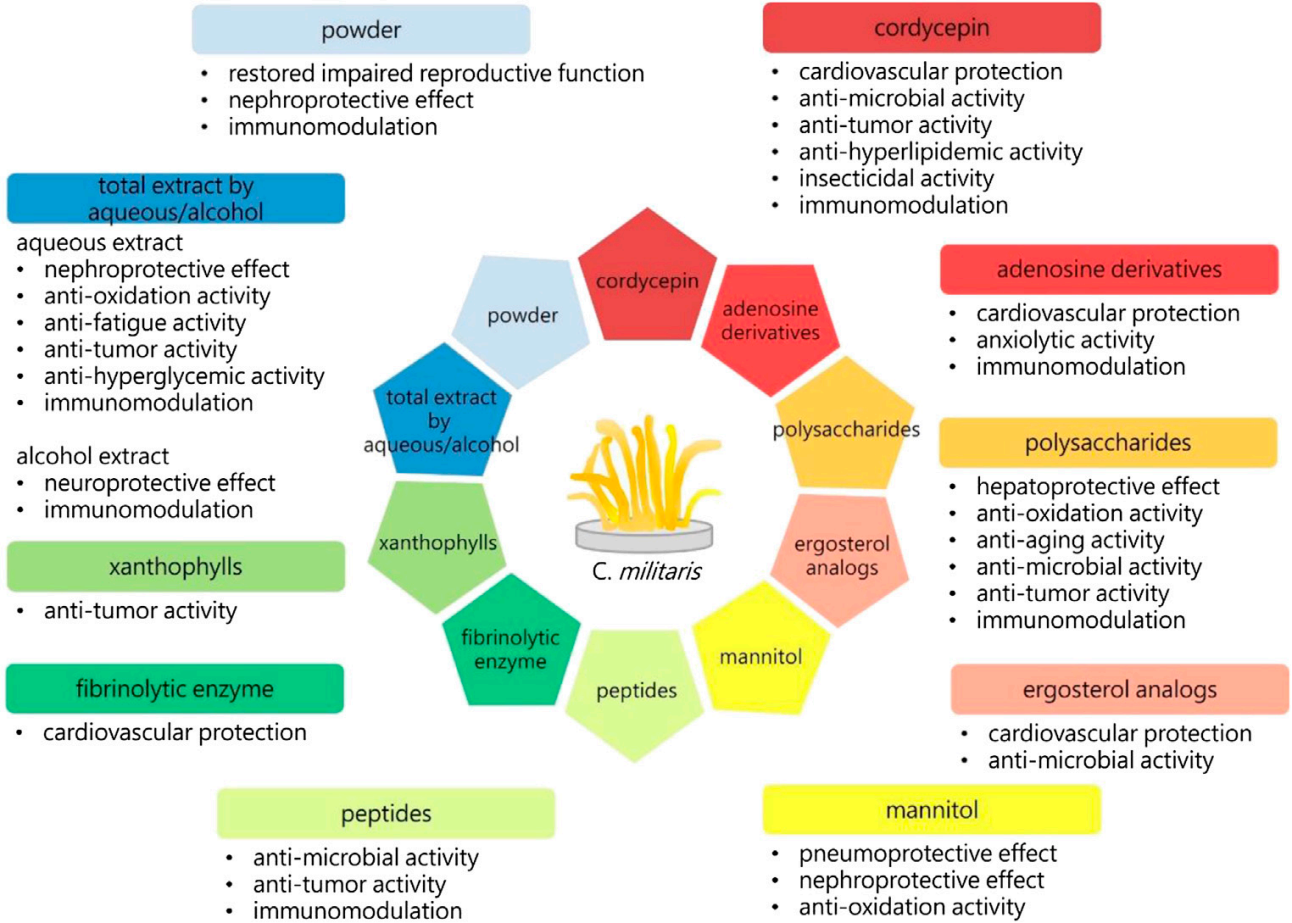
The biological activities of *C. militaris*. 3′-deoxyadenosine (cordycepin), adenosine derivatives, polysaccharides, ergosterol analogs, mannitol, peptides, fibrinolytic enzyme, xanthophylls, aqueous or alcohol extracts, and the powders of *C. militaris* have exhibited specific biological activities. The immunoregulation is one of the important functions of *C. militaris*. Immune response has been divided into type 1 and type 2, the balance between these two types of immunity is crucial to our health.

*Cordyceps militaris* (*C. militaris*), belonging to the family *Clavicipitaceae*, is a fungus with a long history of widespread use in folk medicine (Figure 1) (Zheng et al., 2011; Wang et al., 2018; Yoon et al., 2018; Qin et al., 2019; Zhang et al., 2019; Zhang et al., 2019; Xiang et al., 2020). Although *C. militaris* is found in East Asia, *C. militaris* is difficult to find in the wild due to its rarity. Thus, many investigators have been devoted to focused on the artificial cultivation of *C. militaris* using cutting-edge technology (Cui, 2015). Many biological and pharmacological functions of this fungus have been identified, such as its antioxidative, antihyperglycemic, antitumor, and immunomodulatory effects (Won and Park, 2005; Dong et al., 2013; Shen et al., 2017; Meng et al., 2018; Yoon et al., 2018; Qin et al., 2019; Sun et al., 2019; Zhang et al., 2019; Guo et al., 2020; Khan and Tania, 2020; Zhang et al., 2020a; Zhang et al., 2020c). In addition, our group has demonstrated that cultivate *C. militaris* can induce apoptosis and autophagy in human glioblastoma cells (Lin et al., 2011; Yang et al., 2012). Numerous review articles have focused on the biological activities of *C. militaris*, some of which are summarized in Table 1 and Figure 2 (Das et al., 2010; Lin and Li, 2011; Yue et al., 2013; Liu et al., 2016a; Liu et al., 2016b). In addition, some of the active compounds identified in this fungus have been tested in preclinical trials and clinical trials (Sun et al., 2014; Kang et al., 2015; Sun et al., 2019; He et al., 2020).

**TABLE 1:** Summary of the biological activities of *C. militaris*.

Title	Brief descriptions	References
Biotechnological production and applications of *Cordyceps militaris*, a valued traditional Chinese medicine	1. Antibacteria, antiinflammation, antiplatelet aggregation, antihyperlipidemic and antitumor activities were found in cordycepin	(Cui, 2015)
	2. Chronic heart failure, immunomodulatory effect, sleep-wake regulation, vessels tone regulation, antidepressant, anticonvulsant, amnesic and anxiolytic activities were found in adenosine derivatives	
	3. Antiviral effect, antitumor, immunomodulation, antioxidative and antiaging activities were found in polysaccharide	
	4. Antiviral, antiarrhythmic and blocking the activated human mesangial cells activities were found in ergosterol analogs	
	5. Diuretic, antitussive and anti-free radical activities were found in mannitol	
	6. Antitumor, immunomodulation and antifungal effects were found in peptides	
	7. Antithrombosis effect was found in fibrinolytic enzyme	
	8. Antitumor effect was found in xanthophylls	
Pharmacological actions of *Cordyceps*, a prized folk medicine	1. Antiinflammatory effect was found in polysaccharides	(Ng et al., 2005)
	2. Antioxidant, inhibiting tumor growth and antiangiogenesis, reducing hyperglycemia and inhibiting low-density lipoprotein-induced glomerulosclerosis activities were found in water extract of *C. militaris*	
Medicinal uses of the mushroom *Cordyceps militaris*: Current state and prospects	1. Improved sperm production, modulated immune response, prevented oxidation, antitumor activity, antimicrobial activity, insecticidal activity, larvicidal activity, antifibrotic effect, hypoglycemic effect, antihyperlipidemic effect, antiangiogenesis activity, antidiabetic effect, antifatigue activity, neuroprotection, liver protection, renoprotection and pneumo-protection activities were found	(Das et al., 2010)
*Cordyceps* as an herbal drug	1. Antitumor effect, inducing dendritic cells maturation, increasing IL-18 gene expression, improving insulin resistant and insulin secretion and increase physical strength were found in aqueous extract of *C. militaris*	(Lin and Li, 2011)
	2. Antiinflammation effect, improved and restored impaired reproductive function and renal protection were demonstrated by direct administration of *C. militaris* powder	
The genus *Cordyceps*: a Chemical and pharmacological review	1. Immunomodulatory function, antitumor activity, nephroprotective activity, hepatoprotective activity, neuroprotective effect, antioxidative effect, antimicrobial effect, insecticidal activity, antifibrotic effect and hypoglycemic activity were found	(Yue et al., 2013)

**FIGURE 3:**
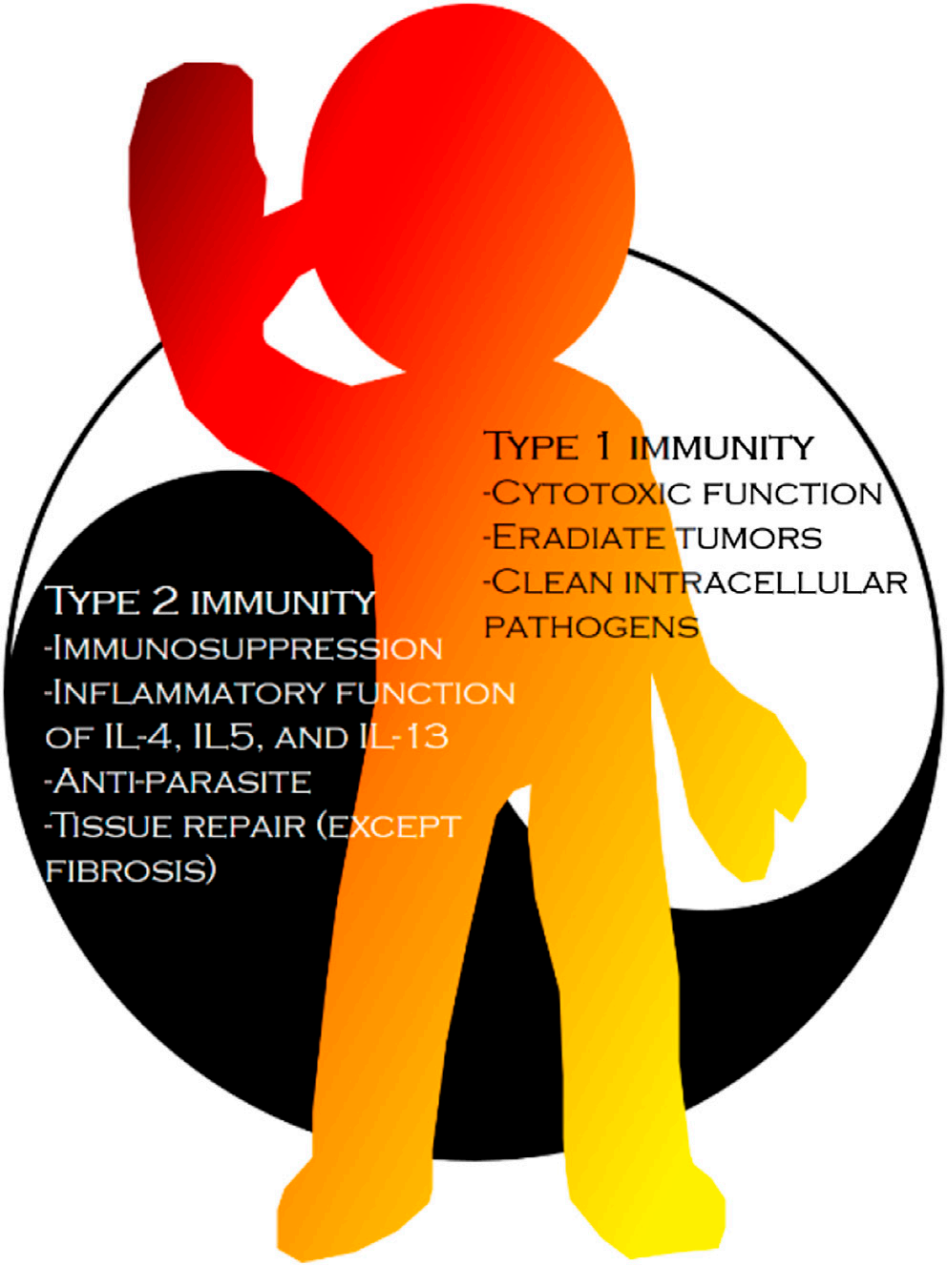
The difference between type 1 immunity and type 2 immunity. Type 1 immunity is related to cytotoxic functions and type 2 immunity is related to immunosuppressive function or inflammatory functions of cytokine IL-4, IL-5 and IL-13. In nowadays, type 1 immunity is associated with tumor and intracellular pathogens killing and type 2 immunity is associated with extracellular parasite eradiation and tissue repair, except fibrosis.

However, few review papers have focused on the relationships between the bioactive compounds of *C. militaris* and immunomodulation, despite the importance of this topic (Zhang et al., 2020b). The balanced interplay between type 1 and type 2 immunity is crucial to health (Bieber, 2008; Kay, 2001a, Kay, 2001b; Lloyd and Snelgrove, 2018, McInnes and Schett, 2011; Moutsopoulos et al., 2017). For example, autoimmune diseases (e.g., rheumatoid arthritis) are related to stronger type 1 immunity, and atopic diseases (e.g., asthma, atopic dermatitis, and allergic rhinitis) are associated with stronger type 2 immunity. This two fold immunity classification can be considered analogous to the “Yin–Yang” in traditional Chinese medicine (Figure 3). Generally, immune response can be divided into type 1 and type 2 immunity (i1–i2 axis). The i1–i2 axis in helper (Th) T cells represents Th1 and Th2, while the i1–i2 axis in macrophages represents M1 and M2 (Lozupone, 2018; Shapouri-Moghaddam et al., 2018). Type 1 immunity is related to cytotoxic functions including the activities of natural killer (NK) cells, Th1, and CD8^+^ T cells, and its major function in hosts is to eradicate tumor cells or cells with intracellular pathogens. By contrast, type 2 immunity is related to immunosuppressive functions—TGF-*β* and interleukin (IL)-10 are common markers—and the inflammatory functions of cytokines IL-4, IL-5, and IL-13; the major function of type 2 immunity is antiparasitic activity, such as the activation of eosinophils and mast cells as well as the secretion of IgE by B cells (Yamaguchi et al., 2015). Type 1 immunity can cause much greater tissue damage than type 2 immunity, and many researchers have claimed that type 2 immunity is crucial in tissue repair (Gause et al., 2013). However, in some cases, type 2 immunity may also cause tissue damage, such as tissue fibrosis (Gieseck et al., 2018).

In the current review, a survey was conducted to update on research activities related to immunology exhibited by *C. militaris* which is divided into three sections, each of which addresses a bioactive form of *C. militaris*: total extracts, polysaccharides and 3′-deoxyadenosine (cordycepin). Each bioactive compound of *C. militaris* was classified into either the type 1 or type 2 immune response category. This article is the first to classify the immunomodulatory effects of different extracts of *C. militaris*. In addition, this review can provide the readers a comprehensive understanding on the immunomodulatory effects of the precious folk medicine and guidance on having it for both health and immune deficient people.

## Immunomodulatory Functions of *C. militaris*

### Total Extracts

To understand the pharmacological effects of *C. militaris* on immune regulation, researchers have used various extraction methods to test its biological functions (Lu et al., 2014; Wang and Wang, 2017), and total extracts are one commonly used method (Chu et al., 2011). Some findings of related studies are summarized in Table 2. For example, the anti-inflmmatory activity of the extracts from fruiting body and cultured mycelia of *C. militaris* was compared by Won and Park (2005) (Won and Park, 2005). Two different parts of the fungus were extracted using 70% ethanol. Results indicated that the extracts from both the fruiting body and cultured mycelia could reduce ear edema in an animal study. The inhibition percentages of fruiting body extracts, cultured mycelia extracts, and indomethacin were 58.7, 51.8, and 61.6%, respectively. The two extracts were then further used to examine the inducible nitric oxide synthase (iNOS) expression, which is highly expressed in inflammatory diseases and leads to cellular injury. The results revealed that fruiting body extracts and cultured mycelia extracts had similar antiinflammatory effects. Further investigation indicated that fruiting body extract and cultured mycelia extract could substantially reduce vascular development and reduce angiogenesis, with stronger antiangiogenesis effects observed in the cultured mycelia extracts.

**TABLE 2:** The immunomodulation effects of total plant extracts from *C. militaris*.

Origin	Extracting Method	Function and Possible Mechanism	References
Fruiting body extract and cultured mycelia extract	70% ethanol at room temperature	1. Inflammation ↓ (fruiting bodies extract ≈ cultured mycelia extract) ↑	(Won and Park, 2005)
		2. iNOS expression ↓ (fruiting bodies extract ≈ cultured mycelia extract)	—
		3. Angiogenesis ↓ (cultured mycelia extract > fruiting bodies extract)	—
Fruiting body extract	Distilled water and then boiled for 4 h	1. IL-18 ↑ expression in mice brain and liver tissue	(Kim et al., 2008)
		2. IL18 ↑ and INF-*γ* ↑ in RAW 264.6 macrophage (INF-*γ* enhancement might be associated with IL-18 pathway)	
Fruiting body extract	Distilled water and then boiled for 30 min	In OVA-induced asthma mice	(Hsu et al., 2008)
		1. Serum IgE↓ in OVA-induced asthma mice	
		2. Infiltrated inflammatory cells↓ and thickening of smooth muscle layer in airway ↓	
Fruiting body extract	Distilled water and 60 °C for 6 h	● macrophage activity modulation	(Shin, Kwon, et al., 2010; Shin, Park, et al., 2010)
		1. Macrophage proinflammatory cytokine ↑ (such as IL-1*β*, IL-6, TNF-*α*, and PGE_2_); iNOS and COX-2 expression ↑	
		2. B7-1/-2 and ICAM-1 expression ↑	
		3. Might be related to NF-*κ*B pathway	
		● dendritic cells activity modulation	
		1. *MHC* class *I* and *MHC* class *II* molecule expression ↑, but do not alter the phagocytotic effects	
		2. *MHC* class *I* and *MHC* class *II* presentation↑, which can help CD4 T cells and CD8 T cells activation ↑	
		3. Inflammatory cytokine expression ↑ (such as IL-1*β*, IL-6 and TNF-*α*); iNOS and COX-2 expression ↑	
		4. Might be related to NF-*κ*B pathway	
Mycelium extract	Distilled water and then boiled for 48 h	● in dextran sodium sulfate-induced acute colitis mice model	(Han et al., 2011)
		1. Reducing body weight loss, diarrhea and gross bleeding	
		2. Preventing shortening of colon length and crypt length	
		3. Suppressing epithelial damage, loss of goblet cells, loss of crypts and infiltration of inflammatory cells	
		4. iNOS and TNF-*α* expression ↓ in colon tissue	
		5. Mast cells degranulation ↓ in colon tissue	
		● in cell line model	
		1. iNOS and TNF-*α* expression ↓ in LPS-induced RAW 264.7 macrophage	
		2. Degranulation of antigen-stimulated mast cells ↓	
Mycelium extract	The total extract was dissolved with water, after remove the insoluble solid by filtration, the liquid phase was extracted by hexane, ethyl acetate, or tert-butyl alcohol	1. Degranulation of antigen-induced mast cells ↓	(Oh et al., 2011)
		2. Reduction passive cutaneous anaphylaxis reaction in antigen-induced mice model	
		3. The antiallergic effects might be associated with lowing the expression level of the phosphorylation syk, ERK, p38 and JNK.	
Fresh *C. militaris* and dried *C. militaris*	With water and the mixture was homogenated at 4,000 r/min for 15 min, then treated with ultrasonic waves at 30°C for 30 min	1. Reversing the reduction of spleen and thymus index induced by cy (fresh *C. militaris* > dried *C. militaris*)	(Zhu et al., 2013)
		2. Increasing the phagocytotic effect in cy-induced mice (fresh *C. militaris* > dried *C. militaris*)	
		3. Recovering the IL-2 and IFN-*γ* secretion level in cy-injected mice or cy-treated T lymphocytes (fresh *C. militaris* > dried *C. militaris*)	
		4. The chemical composition polysaccharides, total polyphenol and total flavonoids were greater in fresh *C. militaris* than dried *C. militaris*; adenosine and cordycepin in fresh *C. militaris* and dried *C. militaris* were nearly the same	
*C. militaris* extract	Provided by dong-a pharm. Co., LTD. (korea)	● in health mice	(Lee et al., 2014)
		IL-12 ↑ in plasma and NK cells population ↑ in peripheral blood	
		● in H1N1 infected mice model	
		1. mortality ↓	
		2. IL-12 ↑ and TNF-*α* ↓	
Dried *C. militaris*	50% ethanol at room temperature and at normal pressure for 3 days	1. IL-2 ↑ and IFN-γ ↑	(Kang et al., 2015)
		2. Enhancing NK cells activity and T-cell proliferation	
*C. militaris* grown on germinated soybean extract (called GSC)	80% EtOH for 48 h twice	● in DNFB-induced contact dermatitis mice model	(Park, 2015)
		1. Ear swelling ↓	
		2. Reducing T cells infiltrated ↓ (including CD4 and CD8)	
		● in concanavalin-A stimulated T cells proliferation model	
		1. T cells proliferation ↓	
Fruiting body extract	Distilled water at 65°C for 2 h (pH 6.5)	1. Increasing thymus and spleen index	(Liu et al., 2016a)
		2. Total white blood cell count were increased (lymphocytes ↑; monocytes ↑; neutrophils ↓; eosinophils and basophils no changed)	
		3. Increasing IgG content	
		4. Increasing TNF-*α* and IL-1*β* expression in spleen	
		5. Increasing the activity of superoxidase dismutase, glutathione peroxidase, and total antioxidant capacity activity in liver, kidney and heart tissue; MDA level were decreased in liver, kidney, heart and serum sample	

Water extract from the fruiting body of *C. militaris* was prepared and orally administered to mice at a dose of 20 mg/kg (Kim et al., 2008). Kim et al. (2008) reported that the extracts could increase the level of interferon (IFN)-γ secretion by macrophages through IL-18. Therefore, they proposed that *C. militaris* could be used as an immune activator or antitumor agent.

Water extracts were obtained from fruiting body of *C. militaris* by Hsu et al. (2008) and found to have potential therapeutic effects on asthma in an ovalbumin (OVA)-induced asthma mice model (Hsu et al., 2008). Briefly, the mice were given 100 µg of OVA through intraperitoneal (IP) injection on day 0, and subsequently, 50 µg of OVA was administered intranasally on days 14, 25, 26, and 27 to induce asthma. A total of 4 g/kg/day of *C. militaris* extract was administered orally to treat the mice on days 15–27. Results revealed that the serum immunoglobulin E (IgE) level decreased, but not significantly, in the group treated with *C. militaris* extract compared with nontreated control, montelukast (leukotriene receptor antagonist) and prednisolone sodium phosphate solution (steroid) groups. The serum IgE levels in the nontreated control, steroid-treated, montelukast-treated and *C. militaris* extract-treated mice were 1833.52 ± 402.03, 574.03 ± 120.71, 284.46 ± 45.41 and 1,591.35 ± 350.68 ng/ml, respectively. In addition, the researchers observed fewer infiltrating cells in the airway in *C. militaris* extract-treated group than in the nontreated control, montelukast and steroid groups. However, the difference was not significant. Infiltrating cell counts in the nontreated control, steroid-treated, montelukast-treated and *C. militaris* extract-treated groups were 11.83 ± 2.2 × 10^5^/ml, 3.83 ± 0.6 ×10^5^/ml, 4.33 ± 0.85 × 10^5^/ml, and 8.5 ± 1.71 × 10^5^/ml, respectively. Finally, histology was used to evaluate airway inflammatory changes. The results indicated that *C. militaris* extract treatment only partially attenuated the inflammatory cells recruited in the airway and the smooth muscle layer thickened in the OVA-treated group. The researchers concluded that *C. militaris* extract could modulate airway inflammation in asthma but was only suitable for use as an auxiliary agent in asthma control because its effect was not as strong as those of montelukast and steroids.

Water extracts from the fruiting body of *C. militaris* were demonstrated by Kim’s group (2010) that could modulate macrophage activity (Shin et al., 2010). They used 12.5, 25, 50, 100, or 200 μg/ml extracts to treat RAW 264.7 macrophages or primary culture peritoneal macrophages. They observed that expression of nitrogen oxide; proinflammatory cytokines, such as IL-1*β*, IL-6, tumor necrosis factor-*α* (TNF-*α*), and prostaglandin E2 (PGE_2_); and iNOS and cycloxygenase-2 (COX-2) were significantly elevated in a dose-dependent manner compared with nontreated controls. In addition, the expression of costimulatory molecules such as B7-1 [e.g., cluster of differentiation (CD)80] protein, B7-2 (e.g., CD86) protein, and intracellular adhesion molecules-1 (ICAM-1) were also enhanced. The researchers reported that *C. militaris* extracts could activate macrophage activity through activation of nuclear factor kappa-light-chain-enhancer of activated B cells (NF-*κ*B). In the same year, Kim’s group also reported that the water extracts from the fruiting body of *C. militaris* could modulate dendritic cell function (Shin et al., 2010). They used 12.5, 25, 50, 100, or 200 μg/ml extracts to treat both dendritic cells DC2.4 and primary bone marrow-derived dendritic cells. They found that *C. militaris* extracts could increase the expression of the *H-2K* ^*b*^ gene [major histocompatibility complex (MHC) class I] and *I-A* ^*b*^ gene (MHC class II) but did not alter the phagocytotic activities of dendritic cells. Results further demonstrated that these extracts could enhance the presentation of both MHC class I and MHC class II in dendritic cells, which could successfully increase CD4 T cell and CD8 T cell activation. In addition, the researchers speculated that *C. militaris* extracts could increase inflammatory cytokine expression (such as IL-1*β*, IL-6, and TNF-*α*), and expression of cytokine-related enzymes (such as iNOS and COX-2) and that the activation of dendritic cells caused by *C. militaris* extract might be result from NF-*κ*B upregulation.

Han et al. (2011) found that the water extract of mycelium of *C. militaris* attenuated dextran sodium sulfate–induced acute colitis in mice (Han et al., 2011). They used 500 mg/kg of the extract to treat mice with acute colitis and observed that it could reduce body weight loss, diarrhea, and major bleeding. In addition, *C. militaris* extract could prevent the shortening of the colon length and crypt length and suppress epithelial damage, loss of goblet cells, loss of crypts, and infiltration of inflammatory cells. The researchers revealed that the extract could inhibit iNOS and TNF-*α* expression in the colon of mice with acute colitis and in lipopolysaccharide (LPS)-induced RAW 264.7 macrophages. Degranulation of mast cells in colon tissue and antigen-stimulated mast cells could also be suppressed after extract treatment. They exhibited that *C. militaris* extract could be a potential reagent for the prevention or treatment of inflammatory bowel diseases.

Ethyl acetate extract of *C. militaris* reported to inhibit allergy reaction by Oh et al. (2011) (Oh et al., 2011). They used 10, 30, 100, or 300 μg/ml extracts to examine the antiallergic response in RBL-2H3 mast cells and found that ethyl acetate extracts could inhibit antigen-induced degranulation with half maximal inhibitory concentration (IC_50_) = 28.5 μg/ml in RBL-2H3 cells. The inhibitory effect of the extract on antigen-induced passive cutaneous anaphylaxis was evaluated in a mice model. Results indicated that the ethyl acetate extract could reduce the passive cutaneous anaphylaxis reaction in a dose-dependent manner with median effective dose (ED_50_) = 665 mg/kg. Furthermore, the extract could reduce TNF-*α* and IL-4 expression in antigen-stimulated mast cells. This reaction might be associated with lowering the expression levels of the phosphorylation tyrosine-protein kinase (SYK), extracellular-signal-regulated kinase (ERK), p38 mitogen-activated protein kinases (p38), and c-Jun *N*-terminal kinase (JNK). Results also revealed that the ethyl acetate extract could directly mediate mast cell immune response.

Zhu et al. (2013) compared the immunomodulation effects of fresh and dried *C. militaris* extracts (Zhu et al., 2013). Briefly, 50, 100, or 200 mg/kg of fresh or dried extracts were used to treat cyclophosphamide (cy)-induced immunosuppression in mice. Analysis results indicated that the amount of adenosine and cordycepin in fresh and dried *C. militaris* was almost identical. By contrast, polysaccharides, total polyphenol, and total flavonoids were more abundant in fresh than in dried extracts. The researchers observed that fresh or dried *C. militaris* could reverse the inhibition of spleen and thymus index (spleen or thymus weight/body weight) in cy-induced mice in a dose-dependent manner. In addition, both fresh and dried *C. militaris* could recover the phagocytotic effect in cy-treated mice, and the fresh extract had a stronger effect than did the dried extract. Both fresh and dried *C. militaris* could considerably increase IL-2 and IFN-*γ* secretion levels in cy-induced mice or cy-treated T cells. The researchers concluded that fresh *C. militaris* is superior to dried *C. militaris* in terms of immune modulation.

*C. militaris* extract was reported by Lee et al. (2014) to have some protective effects in influenza A virus-infected mice (Lee et al., 2014). After 7 days of oral administration of 30, 100, or 300 mg/kg/day *C militaris* extracts, mice were then infected with influenza A/NWS/33 (H1N1) virus. The researchers found that IL-12 was elevated and TNF-*α* was reduced. They argued that the protective effect of the extract in H1N1-infected mice might be related to the elevation of IL-12, increase of natural killer (NK) cells, and decrease of TNF-*α* level.

An ethanol extract of *C. militaris* was exhibited by Kang et al. (2015) to enhance cell-mediated immunity in healthy Korean men (Kang et al., 2015). A total of 1.5 g/day of *C. militaris* was orally delivered to healthy volunteers. Results revealed that the extract did not cause any adverse reaction. The researchers found that the IL-2 and the IFN-*γ* were significantly increased after the 4 weeks treatment (N = 39) compared with the placebo group (N = 40). In addition, they observed that *C. militaris* could increase NK cell activity and T cell proliferation. They posited that the extract could serve as a safe immunomodulator to increase cell-mediated immunity.

*C. militaris* grown on germinated soybean extract (GSC) could inhibit 2,4-dinitro-1-fluorobenzene (DNFB)-induced contact dermatitis (Park, 2015). 300 mg/kg of GSC was used to treat DNFB-induced contact dermatitis and it was found that GSC mitigated ear swelling compared with results in the nontreated control. Furthermore, immunohistochemical analysis of the infiltrated T cells in the ear tissue of the mice illustrated that GSC could significantly reduce CD4 and CD8 T cell infiltration in the ear of DNFB-induced contact dermatitis mice. In addition, they found that GSC could inhibit concanavalin A–induced T cell proliferation. They suggested that GSC could act as a reagent to alleviate allergic contact dermatitis.

Extracts from fruiting body of *C. militaris* exhibited an immunomodulatory and antioxidative activity (Liu et al., 2016a; Liu et al., 2016b). Three dosages of extracts (50, 100, or 200 mg/kg/day) were fed to healthy Kunming mice. The researchers demonstrated that thymic and splenic indices were significantly increased in mice treated with *C. militaris* polysaccharides in a dose-dependent manner. Total white blood cell count was increased in extract-treated mice in a dose-dependent manner. In the white blood cell population, lymphocytes and monocytes were increased, neutrophils were decreased, and eosinophils and basophils seemed to display no overt changes. In addition, the IgG content increased in the mice treated with *C. militaris* polysaccharides. TNF-*α* and IL-1*β* expressions were also increased in the spleen after treatment with the extract. Superoxidase dismutase, glutathione peroxidase, and total antioxidant capacity activity were increased in liver, kidney, and heart tissue; the end product of lipid peroxidation, the malondialdehyde levels, was decreased in the liver, kidney, heart, and serum samples. They concluded that *C. militaris extracts* could modulate the immune system and antioxidative enzymes in heathy organisms.

The results presented in this section lead to some conclusions. First, nearly no difference exists between the extracts of fruiting body and those cultured mycelia in terms of immunomodulatory effect. However, cultured mycelia extract exhibited a more potent effect in antiangiogenesis than did the fruiting body extract. Second, in comparison with dry *C. militaris*, the fresh extract had a stronger immunoregulation effect, which could be attributed to the higher levels of polysaccharides, total polyphenol, and total flavonoids in fresh *C. militaris* (Zhu et al., 2013). Finally, the total extracts of *C. militaris* using water or 50% ethanol are likely to activate macrophage and dendritic cells while reducing allergy effects. Furthermore, *C. militaris* extracts using 70–80% ethanol seems to have anti-inflammatory effects.

### Polysaccharides

Polysaccharides are crucial bioactive components in *C. militaris* (Beltrame et al., 2019; Yang et al., 2019; Barcelos et al., 2020; Mohan et al., 2020; Wu et al., 2020). Polysaccharides are known to have antiviral, antioxidative, antiinflammatory, antitumor, neuroprotective, antihypertensive, and immunomodulatory biological effects (Sims et al., 2011; Lee et al., 2017; Rodrigues et al., 2020). Some research findings related to polysaccharides are summarized in Table 3.

**TABLE 3:** The immunomodulation effects of polysaccharides from *C. militaris*.

Name	Size and Structural Content	Function and Possible Mechanism	References
CPS-1	1. MW = 2.3 × 10^4^ Da	1. Reduced inflammation	(Yu et al., 2004)
	2. Content: Rha, Xyl, D-Man, D-Glc and D-Gla in a ratio of 1:6.43:25.6:16.0:13.8	2. Modulate humoral immunity	
	3. Structure: Mannose bonded by (1→2) linkage, xylose bonded by (1→4) linkage, and rhamnose bonded with galactose by (1→2) or (1→3) linkage	3. No effect on phagocytotic function on innate immunity	
		4. No effect on cellular immunity	
Cordlan	1. MW = 5.76 × 10^5^ Da	**●** antiinfluenza a virus effect by immunomodulation	(Ohta et al., 2007; Kim et al., 2010)
	2. Content: D-gal, ara, Xyl and rha in a ratio of 9.11:4.34:1.17:1	1. Prevent influenza a virus infected mice from death	
	3. Structure: Ara*f*-(1→, →5)-ara*f*-(1→, f→4)-gal*p*-(1→ and →4)-gala*p*-(1→residues	2. Increase inflammatory cytokine (IL-1*β*, IL-6, TNF-*α*, IFN-*γ*) and iNOS expression which might increase anti-virus activity	
		3. Increase antiinflammatory cytokine (IL-10) expression which might prevent macrophage excessive activation	
		**●** stimulating dendrtic cells maturation	
		1. Elevating the mature DC phenotypic markers such as CD40, CD80, CD86, MHC-I, and MHC-II molecules and mature DC functional marker such as IL-12, IL-1*β*, TNF-*α* and IFN-*αβ*, enhanced allogenic T cell stimulation, and decreased endocytosis	
		2. Induce immature dendrtic cells into mature dendrtic cells through TLR4 pathway	
Cordysinocan	1. MW = 8.2 × 10^4^ Da	1. Enhancing T lymphocytes proliferation and IL-2 secretion	(Cheung et al., 2009)
	2. Content: D-Glc, D-Man and D-Gal in a ratio of 2.4:2:1	2. Promoting macrophage phagocytotic and acid phosphatase activities	
	3. Structure: Not available	—	
CPSN Fr II	1. MW = 3.6 × 10^4^ Da	1. Elevating TNF-*α* and IL-1*β* secretion in macrophage	(Lee et al., 2015; Lee, Kwon, Won, et al., 2010)
	2. Content: D-Man, D-Gal and D-Glc in a ratio of 10.64:4.69:1; proteins, hexosamine and uronic acid of this polysaccharide are 0.20, 0.06 and 0.29%, respectively	2. Increasing nitric oxide production in macrophage	
	3. Structure: The backbone of (1→2)-linked d-mannopyranosyl and (1→6)-linked d-mannopyranosyl residues, which occasionally branches at O-6. The branches were mainly composed of (1→4)-linked d-galactopyranosyl residues, and terminated with d-galactopyranosyl residues, with a degree of branching of 0.2	3. The macrophage activation might be associated with MAPKs and NF*κ*B *via* TLR2, TLR4 and dectin-1	
CPMN Fr III	1. MW = 2.1 × 10^5^ Da	1. Elevating TNF-*α* and IL-1*β* secretion in macrophage	(Lee, Kwon, Yun, et al., 2010)
	2. Content: D-Man, D-Gal and D-Glc in a ratio of 7.88:2.03:1; proteins, hexosamine and uronic acid of this polysaccharide are 0.21, 0.12 and 0.33%, respectively	2. Increasing nitric oxide production in macrophage	
	3. Structure: The backbone of (1→6)-linked d-mannopyranosyl and (1→6)-linked d-glucopyranosyl residue. The branches were mainly composed of (1→4)-linked d-mannopyranosyl residue, and terminated with d-galactopyranosyl residues and d-mannopyranosyl residues, with a degree of branching of 0.33		
Polysaccharides (CM)	1. MW = 1.27 × 10^5^ Da	1. Elevating the expression of ROS, nitric oxide, TNF-*α* and phagocytic uptake	(Lee et al., 2011)
	2. Content: Not available	2. The immune modulation effects might be associated with NF-*κ*B and all three MAPKs pathways through TLR2 and dectin-1	
	3. Structure: (1→4) or (1→2) linked d-glucopyranosyl or d-galactopyranosyl residue with a (1→2) or (1→6) linked d-mannopyranosyl, d-glucopyranosyl, or d-galactopyranosyl residue as a side chain	3. Antitumor effect by regulating host immune response but not killing the tumor cells directly	
Polysaccharides (CMP)	1. MW = not available	1. Reversing the decreasing of spleen and thymus indices in cy-injected mice	(Wang et al., 2012)
	2. Content: Not available	2. Increasing the lymphocyte proliferation in cy-injected mice	
	3. Structure: Not available	3. Recovered the phagocytotic index in cy-injected mice	
		4. Improving the antioxidative ability in cy-injected mice	
CMP-W1	CMP-W1	1. Inducing mice splenocytes proliferation	(Luo et al., 2017)
	1. MW = 3.66 × 10^5^ Da	2. With synergistical effect on concanavalin-A induced T-cells proliferation	
	2. Content: D-Man: D-Glc: D-Gal with the molar ratios of 2.84:1:1.29	3. With synergistical effect on LPS induced B-cells proliferation	
	3. Structure: Not available	—	
CMP-S1	CMP-S1		
	1. MW = 4.6 × 10^5^ Da		
	2. Content: D-Man: D-Glc: D-Gal with the molar ratios of 2.05:1:1.09		
	3. Structure: Not available		
CMP-III	1. MW = 4.796 × 10^7^ Da	1. Enhancing macrophage phagocytosis ability	(He et al., 2020)
	2. Content: D-Glc: D-Man: D-Gal with the molar ratio of 8.09:1:0.25	2. Increasing TNF-α, IL-6 and NO secretion	
	3. Structure: 1→4)-α-D-Glc (70.08%), 1→4,6)-α-D-Man (9.59%), 1→)-α-D-Man (10.79%) and 1→2,6)-α-D-Gal (3.93%)		
CMPB90–1	1. MW = 5.8 × 10^3^ Da	1. Converting M2 macrophage into M1-like which could increase our body to eradicate tumor	(Bi et al., 2020)
	2. Content: D-Gal: D-Glc: D-Man with the molar ratios of 3.04:1:1.45	2. Increasing T cells activity by inhibiting the PD-L1/PD-1 axis between tumor associated macrophage and T cells	—
	3. Structure: (1→6)-α-d-glucopyranosyl and (1→3)-α-d-glucopyranosyl residues, with branching at O-6, which consists of (1→4)-β-d-mannopyranosyl and (1→6)-α-d-glucopyranosyl residues, respectively	—	—

CPS-1 extracted from *C. militaris* acts as an immunomodulator (Yu et al., 2004). CPS-1 is a polysaccharide with a molecular weight (MW) of 2.3 × 10^4^ Da and is composed of rhamnose (Rha), xylose (Xyl), d-mannose (D-Man), d-glucose (D-Glc), and d-galactose (D-Gal). CPS-1 might contain rhamnose bonded with galactose by a (1→2) or (1→3) linkage, xylose bonded by a (1→4) linkage and mannose bonded by a (1→2) linkage. First, the researchers found that an IP injection of 50, 100, or 200 mg/kg CPS-1 in croton oil–induced mice ear edema could significantly reduce inflammation (the inhibition percentage was 48.3, 61.5, and 71.7%, respectively) in a dose-dependent manner. Second, they injected mice with 25, 50, or 100 mg/kg CPS-1 subcutaneously and found that CPS-1 decreases vascular permeability in the acetic acid–induced model compared with normal saline treatment. Third, the researchers used sheep red blood cells and observed reduced serum hemolysin formation in mice that received IP injections of 15 and 30 mg/kg CPS-1. Finally, CPS-1 was used to evaluate the phagocytotic function and delayed-type hypersensitivity *in vivo*; no differences were observed between the CPS-1-treated group and the control group. According to the aforementioned results, the researchers suggested that CPS-1 can reduce inflammation and humoral immunity but reported no significant change in the phagocytotic function of innate immunity and cellular immunity.

An acid polysaccharide (APS) isolated from *C. militaris* was revealed to have antiinfluenza virus activity by Ohta et al. (2007) (Ohta et al., 2007). APS, with an MW of 5.76 × 10^5^ Da, consists of D-Gal, arabinose (*Ara*), Xyl, Rha, and galacturonic acid. After mice infected with H1N1 were treated with 0.1 mg of APS, the researchers found that APS could reduce mortality in comparison with controls (mortality of APS:mortality of control = 18%:70%). In addition, the researchers observed that APS might modulate macrophage activity to protect against influenza virus A infection. Results indicated that APS could increase TNF-*α* and IFN-*γ* expression in influenza A virus–infected mice. These results were also confirmed using RAW 264.7 macrophage cells: iNOS, IL-1*β*, IL-6, and IL-10 expression levels were elevated after RAW 264.7 macrophage cells were treated with APS. Results indicated that APS could modulate not only inflammatory cytokine (IL-1*β*, IL-6, TNF-*α*, and IFN-*γ*) and iNOS expression, which could increase antivirus activity, but also anti-inflammatory cytokine (IL-10) expression, which could prevent excessive macrophage activation and reduce deaths caused by influenza A virus *in vivo* (Ohta et al., 2007).

Cysinocan, a polysaccharide extracted from *C. militaris* and with a MW of 8.2 × 10^4^ Da, is composed of D-Glc, D-Man, and D-Gal (Cheung et al., 2009). Cheung et al. (2009) found that cordysinocan modulated both T lymphocyte and macrophage activities. The researchers observed that cordysinocan induced T cell proliferation [half maximal effective concentration (EC_50_) = 6 μg/ml] and IL-2 secretion (EC_50_ = 8.5 μg/ml). Moreover, IL-2, IL-6, IL-8, and IL-10 were markedly overexpressed. The study also investigated whether the cordysinocan-induced T cell proliferation occurred through the ERK pathway. The researchers revealed that cordysinocan could act as an immunomodulator on T lymphocytes and macrophages.

Cordlan polysachharide was found to induce dendritic cell maturation (Kim et al., 2010). The authors found that 10, 30, or 100 μg/ml of cordlan could help immature dendritic cells to mature. Cordlan enhanced allogenic T cell stimulation, decreased endocytosis, and elevates the phenotypic markers of mature dendritic cells, such as CD40, CD8 and CD86, the main function of *MHC* class *I*; and functional markers of MHC class II molecules and mature dendritic cell, such as IL-12, IL-1*β*, TNF-*α*, and IFN-*αβ*. The researchers revealed that cordlan induced maturation in immature dendritic cells by failing to induce toll-like receptor (TLR)4 knock out from immature dendritic cells into mature dendritic cells, possibly through the TLR4 pathway.

CPSN Fr II and CPMN Fr III were isolated by Hong’s group (2010). CPSN Fr II, with an MW 3.6 × 10^4^ Da was discovered to consist of D-Man (65.12%), D-Gal (28.72%) and D-Glc (6.12%). CPMN Fr III with a MW of 2.1 × 10^5^ Da consisted of D-Man (72.22%), D-Gal (18.61%) and D-Glc (9.17%) (Lee et al., 2010; Lee et al., 2010). The researchers indicated that CPSN Fr II or CPMN Fr III could both induce macrophages to secrete proinflammatory cytokines (TNF-*α* and IL-1*β*) and produce nitric oxide. They evaluated the structure of CPSN Fr II and found that this polysaccharide had a backbone of (1→2)-linked d-mannopyranosyl and (1→6)-linked d-mannopyranosyl residues, which occasionally branches at O-6. The branches were mainly composed of (1→4)-linked d-galactopyranosyl residues and terminated with d-galactopyranosyl residues, with a degree of branching of 0.2. The researchers also examined the structure of CPMN Fr III and found that this polysaccharide had a backbone of (1→6)-linked d-mannopyranosyl and (1→6)-linked d-glucopyranosyl residue. The branches were mainly composed of (1→4)-linked d-mannopyranosyl residue and terminated with d-galactopyranosyl residues and d-mannopyranosyl residues, with a degree of branching of 0.33. In addition, Hong’s group revealed that polysaccharides isolated from C. militaris–stimulated macrophage activity might be associated with the mitogen-activated protein kinase (MAPK) and NF-*κ*B pathway (Lee et al., 2015). Furthermore, the researchers observed that anti-TLR2, anti-TLR4, and antidectin-1 antibodies could abolish the activation effect on polysaccharide-induced macrophages. According to these findings, the authors suggested that the polysaccharides activated the macrophages, possibly through the MAPK and NF-*κ*B pathway using TLR2, TLR4, and dectin-1. Hong’s group reported that polysaccharides might have immunomodulatory and antitumor effects (Lee and Hong, 2011). Polysaccharide could regulate peritoneal macrophages and RAW 264.7 cells to increase the expression of reactive oxygen species (ROS), nitric oxide, TNF-*α*, and phagocytic uptake in a dose-dependent manner. They posited that the polysaccharide-induced immune response might be associated with NF-*κ*B and all three MAPK pathways through TLR2 and dectin-1. They also evaluated the antitumor effects on polysaccharide-treated B16-F10 melanoma tumor–bearing mice and found that either IP injection or oral administration could reduce the tumor growth rate. Furthermore, they used 500 μg/ml of polysaccharide to directly treat B16-F10 melanoma and found that the extract did not cause B16-F10 melanoma death. Therefore, the results indicated that polysaccharides exert antitumor effects through immunomodulation but do not induce tumor cells death directly.

Polysaccharides could abolish cy-induced immunosuppression in a mice model (Wang et al., 2012). They used 80 mg/kg/day cy through IP injection from day 1 to day 3, and 17.5, 35, or 70 mg/kg of polysaccharide was administrated orally from day 4 to day 18. Subsequently, the spleen and thymus indices, lymphocyte proliferation, phagocytic index, and biochemical parameters were measured. Results revealed that the spleen and thymus indices were reduced in the cy-injected group in comparison with those of the nontreated controls. Moreover, in 17.5, 35, or 70 mg/kg polysaccharide-treated cy-injected mice, the spleen and thymus indices were reversed in a dose-dependent manner. The researchers also found that the lymphocyte proliferation rate in polysaccharide-treated cy-injected mice was significantly increased relative to that of cy-injected mice. Furthermore, the phagocytotic index in cy-treated mice was reduced, but it recovered after polysaccharide treatment. The authors demonstrated that polysaccharide could improve the immune and antioxidation function in cy-induced immunosuppressive mice.

Two polysaccharides (CMP-W1 and CMP-S1) were extracted by Luo et al. (2017) (Luo et al., 2017). CMP-W1 and CMP-S1—with MW of 3.66 × 10^5^ Da and 4.6 × 10^5^ Da, respectively—are both heteropolysaccharides that are composed of D-Man, D-Glc, and D-Gal. The researchers found that these polysaccharides could induce splenocyte proliferation in mice, and also had synergistic effects on concanavalin A–induced T cells and LPS-induced B cells, respectively. They suggested that CMP-W1 and CMP-S1 could act as potential immunomodulatory reagents.

A novel homogeneous polysaccharide named CMP-III was discovered by Lin’s group (2020); it was composed of D-Glc, D-Man and D-Gal with an MW of 4.796 × 10^7^ Da. The main linkage types of CMP-Ⅲ consisted of (1→4)-*α*-D-Glc (70.08%) (1→4,6)-*α*-D-Man (9.59%) (1→)-*α*-D-Man (10.79%) and (1→2,6)-*α*-D-Gal (3.93%). They found that CMP-III enhanced macrophage phagocytosis ability and increased TNF-*α*, IL-6 and NO secretion (He et al., 2020).

Discovered by Da Yu et al. (2020), a polysaccharide called CMPB90–1 with an MW of 5.8 × 10^3^ Da suggested to be capable of converting M2 macrophage into M1-like macrophage and eradicating tumor. In addition, they also found that CMPB90–1 could increase T cell activity by inhibiting the programmed death ligand-1 (PD-L1)/programmed death -1 (PD-1) axis between tumor associated macrophage and T cells. CMPB90–1 is composed of (1→6)-*α*-d-glucopyranosyl and (1→3)-*α*-d-glucopyranosyl residues (with branching at O-6) which consists of (1→4)-*β*-d-mannopyranosyl and (1→6)-*α*-d-glucopyranosyl residues, respectively. These results suggested that CMPB-90–1 might have potential as an immune regulator to aid tumor eradication (Bi et al., 2020).

Pattern recognition receptors are critical in innate immunity (Kawai and Akira, 2010; Fitzgerald and Kagan, 2020). Polysaccharides are one of the suitable candidates to bind pattern recognition receptors and may be able to alter immunity (Schnaar, 2016). Here, we found that the immunomodulatory effects of *C. militaris* polysaccharides might be associated with binding TLR2, TLR4, and dectin-1 on immune cells. Notably, APS, a polysaccharide derived from *C. militaris*, was demonstrated to have perform two types of functions, namely immune stimulation and anti-inflammation, in an influenza virus A–infection mice model. This crucial finding suggests that APS can be used for the treatment of microbe-induced cytokine storms, a life-threatening condition caused by excessive immune response (D'Elia et al., 2013).

### Cordycepin

Cordycepin is a valuable bioactive compound produced by *C. militaris* that was discovered in the early 1950s (Cunningham et al., 1950; Ruthes, 2016; Qin et al., 2019). Many researchers have demonstrated the strong immunomodulatory effects of cordycepin (Kitamura et al., 2011; Wang et al., 2019). The findings of its immunomodulatory effects are summarized in Table 4.

**TABLE 4:** The immunomodulation effects of cordycepin.

Dosage	Model	Function and Possible Mechanism	References
5, 10, 15, 30 μg/ml	LPS-induced RAW 264.7 macrophage	1. Inhibiting iNOS, COX-2 and TNF-*α* expression	(Kim et al., 2006)
		2. The inhibition effect might be associated with suppression of NF-*κ*B through akt and p38 inhibition	
5, 10, 20, 40 μg/ml	LPS-induced RAW 264.7 macrophage	1. Decreasing TNF-*α*, IL-6, IL-1*β*, iNOS and COX-2 expression	(Shin et al., 2009)
		2. Reducing T2D associated genes expression, such as RANTES, 11*β*-HSD1 and PPAR*γ*	
		3. Co-stimulatory molecule B7-1/-2 and ICAM-1 were reduced	
		4. The anti-inflammatory and anti-diabetic effects might be related to NF-*κ*B pathway	
5, 10, 20, 40 μg/ml	LPS-induced RAW 264.7 macrophage	1. Decreasing M1 cytokines such as IL-1*β* and TNF-*α* secretion and M1 chemokine and their receptor such as CX3CR1, CX3CL1 and RANTES expression	(Shin et al., 2009)
		2. M2 cytokine such as IL-1ra, IL-10 and TGF-*β* were up-regulated	
1, 2.5, 5, 7.5 μg/ml	LPS-induced BV2 microglia	1. Reducing iNOS and COX-2 expression that result in decreasing the level of nitric oxide and PGE_2_	(Jeong et al., 2010)
		2. Decreasing the secretion level of TNF-*α* and IL-1*β*	
		3. The inhibition of immune response might be associated with NF-*κ*B, akt, and MAPK signaling pathways	
10, 50, 100, 200 μg/ml	TNF-*α*-activated transfected NF-*κ*B luc and empty pcDNA3.1 vector HEK-293 T	1. Reducing IKK*γ* degradation	(Ren et al., 2012)
		2. IKK*γ* degradation result in lowing the activity of IKK*α*/*β*	
		3. Reducing the activity of IKK*α*/*β* could reduce the degradation of I*κ*B*α*	
		4. Presence lots of I*κ*B*α* reducing the activation of NF-*κ*B pathway	
10, 20, 40 μg/ml	Concanavalin A-induced T cells and DNFB-induced delayed-type hypersensitivity reaction	1. Decreasing concanavalin A-induced T cells proliferation	(Xiong et al., 2013)
		2. Reducing both Th1 (IL-2 and IFN-*γ*) and Th2 cytokine (IL-4 and IL-6) secretion in concanavalin A-induced T cells	
		3. Abolishing the DNFB-induced delayed-type hypersensitivity reaction	
		4. The T cells suppression might be associated with NFAT2 signaling pathway	
10, 20, 30 μg/ml	LPS-induced RAW 264.7 macrophage	1. Reducing COX-2 and iNOS expression	(Choi et al., 2014)
		2. Decreasing the expression of IL-1*β* and TNF-*α*	
		3. P65 expression decreased in nucleus and I*κ*B expression increased in cytosol	
		4. Suppression of MAPKs activity	
		5. Abolishing the expression of LPS-induced TLR4 and MyD88	
10, 20, 40 mg/kg	OVA-induced murine model	1. Reducing serum IgE level and BAL fluid eotaxin, ICAM-1 and Th2 cytokine	(Yang et al., 2015)
		2. Decreasing infiltrated cells in BAL fluid, including eosinophils, neutrophils, macrophages and lymphocytes	
		3. Suppression the airway inflammation and mucus secretion	
		4. The mechanism might be associated with p38-MAPK and NF-*κ*B signaling pathway	
1, 5, 10, 20 mg/kg	Traumatic brain injury rat model	1. Neurological severity score, brain infarction volume and brain water content were significantly reduced	(Yuan et al., 2016)
		2. ZO-1 and occludin were restored	
		3. IL-1*β*, iNOS, myeloperoxidase, matrix metalloproteinases-9 and NADPH oxidase-1 were reduced and arginase and IL-10 were increased	
0.1, 1 or 10 µM	TSLP-induced HMC-1 human mast cell	1. Reducing IL-6, TNF-*α*, IL-1*β* and IL-13 expression	(Yoou et al., 2017)
		2. Decreasing pSTAT6 expression and cells proliferation	
		3. Down-regulating murine double minute 2 and Bcl-2 expression and up-regulating p53, caspase 3 and cleaved poly ADP-ribose polymerase levels	
50 mg/kg	With or without budesonide treated OVA-induced asthma rat model	1. Reducing the cytokine IL-5, IL-13 and TNF-*α* expression and IgE levels	(Fei et al., 2017)
		2. Reducing the thickness of bronchial wall	
		3. Up-regulating adenosine A_2A_ receptor and down-regulating p38 MAPK in cordycepin with or without budesonide group but not in budesonide alone group	
3 mg/kg, 5 mg/kg and 12.5 mg/kg	CD-1 mice with anxiety-like behaviors using elevated plus maze or light-dark box tests	1. Reducing mice anxiety	(Gao et al., 2019)
		2. Increasing IL-4 and IL-13 expression	
		3. Reducing TNF-α and IL-1β	
2 mg/kg, 10 mg/kg and 20 mg/kg	CFA induced paw edema	1. Reducing T cell activity and T cell proliferation	(Wang et al., 2020)
		2. Decreasing IL-2 expression	
		3. Inhibiting TCR signaling cascade(by reducing ZAP70 and erk phosphorylation)	

Cordycepin could inhibit iNOS expression in LPS-induced RAW 264.7 macrophage cells (Kim et al., 2006). The researchers treated LPS-induced RAW 264.7 macrophage cells with 5, 10, 15, or 30 μg/ml of cordycepin, which reduced the production of nitric oxide in a dose-dependent manner in comparison with nontreated group. In addition, the authors detected iNOS, COX-2, and TNF-*α* expression in these cells and found that cordycepin could significantly reduce their expression. According to their findings, the inhibition of iNOS, COX-2, and TNF-*α* expression might be due to the suppression of NF-*κ*B through Akt and p38 inhibition.

Kim’s group (2009) found that cordycepin could regulate type II diabetes (Shin, Lee, et al., 2009). The researchers used 5, 10, 20, or 40 μg/ml cordycepin to treat LPS-induced RAW 264.7 macrophage cells and found that TNF-*α*, IL-6, IL-1*β*, iNOS, and COX-2 expression decreased significantly in cordycepin-treated LPS-induced RAW 264.7 macrophage cells compared with that in the nontreated controls in a dose-dependent manner. They tested the type II diabetes–related genes in LPS-induced RAW 264.7 cells and found that LPS could greatly increase the levels of *RANTES*, *11β-HSD1*, and *PPARγ* genes; however, type II diabetes–related genes decreased signifcantly in the cordycepin-treated group. In addition, cordycepin could decrease the expression of costimulatory molecules, such as B7-1/-2 and ICAM-1, in LPS-induced RAW 264.7 cells. The researchers also observed that cordycepin could decrease the expression of NF-*κ*B p65 in LPS-exposed RAW 264.7 macrophage cells. In summary, this study demonstrated that cordycepin has antiinflammatory and antidiabetic effects on macrophages, and that these effects might be related to the NF-*κ*B pathway. In the same year, Kim’s group demonstrated that cordycepin could switch the phenotype of macrophages to M2 macrophage (Shin et al., 2009). They used 5, 10, 20, or 40 μg/ml cordycepin to treat LPS-induced RAW 264.7 cells to analyze the phenotypic switching in macrophage cells. The researchers found that cordycepin reduced the secretion of M1 cytokines, such as IL-1*β* and TNF-*α*, and the expression of M1 chemokine and its receptors, such as CX3CR1, CX3CL1, and RANTES, in LPS-induced RAW 264.7 macrophage cells. M2 cytokines such as IL-1ra, IL-10, and TGF-*β* were upregulated in cordycepin-treated LPS-induced RAW 264.7 macrophages. The authors suggested that cordycepin could induce macrophages to differentiate into M2 macrophages.

Microglia, a type of macrophage present in brain tissue, is instrumental in the host defense and tissue repair in the central nervous system. An abnormal immune response might cause severe neurodegenerative diseases, such as Alzheimer disease, Parkinson disease, multiple sclerosis, trauma, and cerebral ischemia (Sack et al., 2018). Jeong et al. (2010) demonstrated that cordycepin could regulate the immune response of BV2 microglia cells and might, consequently, be a potential therapeutic reagent for neurodegenerative disease (Jeong et al., 2010). When treating LPS-induced BV2 microglia with 1, 2.5, 5, or 7.5 μg/ml cordycepin, nitric oxide and PGE_2_ expression were reduced. In addition, the iNOS, COX-2, TNF-*α*, and IL-1*β* expression levels also decreased in cordycepin-treated LPS-induced BV2 microglia. The researchers also examined whether the inhibition of immune response might be associated with the suppression of the NF-*κ*B, Akt, and MAPK signaling pathways. They argued that cordycepin could modulate microglia cells and could be used to treat neurodegenerative disease. The mechanism by which cordycepin regulated the NF-*κ*B pathway was investigated by Ren et al. (2012) using TNF-*α*-activated transfected NF-*κ*B Luc and an empty pcDNA3.1 vector HEK-293 T (Ren et al., 2012). They found that cordycepin did not block the nuclear translocation of the p65 subunit. Cordycepin inhibited the nuclear factor of the kappa light polypeptide gene enhancer in B cells and alpha (I*κ*B*α*) phosphorylation, reducing I*κ*B*α* degradation. This mechanism might be associated with cordycepin inhibiting of I*κ*B kinase gamma [IKK*γ*, also known as NF-*κ*B essential modifier (NEMO)] degradation and IKK*γ* binding to IKK*α*/*β* to reduce their ability to phosphorylate I*κ*B*α*. The researchers proposed that cordycepin'|’s regulation of the NF-*κ*B pathway might be related to the decreased IKK*γ* degradation, which results in decreasing IKK*α*/*β* activity to phosphorylate I*κ*B*α*.

Cordycepin could supress T cell activation (Xiong et al., 2013). Cordycepin in dosages of 10, 20, or 40 μg/ml could decrease the proliferation of concanavalin A–induced splenocytes in a dose-dependent manner. Results exhibited that cordycepin could reduce both Th1 (IL-2 and IFN-*γ*) and Th2 cytokine (IL-4 and IL-6) secretion. The researchers proposed that cordycepin’s suppression of concanavalin A–induced T cell activation might be attributed to its reduction of phosphorylated I*κ*B levels and nuclear factor of activated T cells 2 (NFAT2). In addition, the DNFB-induced delayed-type hypersensitivity reaction could be reduced in cordycepin-treated mice. They concluded that cordycepin’s suppression of T cell activation *in vitro* and *in vivo* might be associated with the NFAT2 signaling pathway.

The inhibitory effects of cordycepin-treated LPS-induced macrophage activation might be associated with the TLR4-mediated MAPK and NF-*κ*B pathways (Choi et al., 2014). Cordycepin at dosages of 10, 20, or 30 μg/ml were used to treat LPS-induced RAW 264.7 cells, which led to reduced expression levels of iNOS and COX-2. The expression of IL-1*β* and TNF-*α* were also reduced after LPS-induced macrophages were treated with cordycepin. Results indicated that p65 expression decreased in the nucleus and I*κ*B expression increased in the cytosol after pretreating LPS-induced RAW 264.7 cells with cordycepin. The researchers found that MAPK activity could be suppressed in cordycepin-treated LPS-induced RAW 264.7 cells. Furthermore, LPS-induced TLR4 and myeloid differentiation primary response 88 (MYD88) expression was abolished by cordycepin treatment. The authors illustrated that the inhibitory effects of cordycepin on LPS-stimulated RAW 264.7 cells are related to the suppression of the MAPK pathway and the activation of NF-*κ*B by inhibition of the TLR4 signaling pathway.

Cordycepin could alleviate asthma in a mice model through the inhibition of the Th2-type immune response (Yang et al., 2015). In OVA-induced asthma, cordycepin treatment led to a reduction in serum IgE in a dose-dependent manner in comparison with that in the nontreated controls. The researchers observed that eotaxin, ICAM-1, and Th2 cytokines (IL-4, IL-5, and IL-13) in the bronchoalveolar lavage fluid (BALF) were significantly reduced in cordycepin-treated mice. In addition, they found that cordycepin could reduce the total infiltrated cells in the BALF—including eosinophils, neutrophils, macrophages, and lymphocytes. The authors also evaluated the preventive effects of cordycepin on airway inflammation and mucus hypersecretion in a pathological study. The study demonstrated that the aforementioned phenomenon is potentially associated with the blockage of the p38-MAPK and NF-*κ*B signaling pathways.

Cordycepin was reported by Yuan et al. (2016) to protect the integrity of the blood-brain barrier (BBB) in a traumatic brain injury rat model (Yuan et al., 2016). They intravenously injected 1, 5, 10, or 20 mg/kg cordycepin for 30 min to treat traumatic brain injury in rats. The results revealed that the neurological severity score, brain infarction volume, and brain water content were considerably reduced in the cordycepin-treated group in a dose-dependent manner compared with saline-treated rats. The researchers also found that the two major proteins for BBB integrity, ZO-1 and occludin, were restored in rats treated with 20 mg/kg of cordycepin. Furthermore, the study revealed that levels of IL-1*β*, iNOS, myeloperoxidase, matrix metalloproteinases-9, and nicotinamide adenine dinucleotide phosphate oxidase-1 were reduced and those of arginase and IL-10 were increased in cordycepin-treated rats compared with those in the nontreated controls. This research exhibited that cordycepin could serve as a reagent to protect BBB integrity.

Cordycepin was found by Yoou et al. (2017) to be capable of regulate mast cell proliferation (Yoou et al., 2017). Thymic stromal lymphopoietin (TSLP) is a cytokine that is associated with atopic dermatitis. The researchers used 0.1, 1, or 10 µM of cordycepin to treat TSLP-induced human mast cells (HMC-1) and found that cordycepin could reduce IL-13 secretion in a dose-dependent manner. Phosphorylation signal transducer and activator of transcription (pSTAT6) is a well-known protein that is associated with TSLP-induced mast cell proliferation. Results of this study indicated that TSLP-induced HMC-1 cell expression of pSTAT6 could be abolished in the cordycepin-treated group. Furthermore, the researchers indicated that cordycepin could downregulate murine double minute 2 (MDM2) and Bcl-2 expression and upregulate p53, caspase 3, and cleaved poly ADP-ribose polymerase (PARP) levels in TSLP-induced HMC-1 cells. They also revealed that TSLP-induced IL-6, TNF-*α*, and IL-1*β* were reduced in cordycepin-treated TSLP-induced HMC-1 cells. This study successfully demonstrated that cordycepin could reduce IL-13 and MDM2 expression in TSLP-induced HMC-1 cells.

Cordycepin was reported by Fei et al. (2017) to be capable of inhibiting airway remodeling in a chronic asthma rat model (Fei et al., 2017). A dosage of 50 mg/kg of cordycepin with or without budesonide was used to treat rats with OVA-induced asthma to investigate potential underlying molecular mechanisms. The results indicated that cotreatment with cordycepin and budesonide synergistically reduced the total cells, eosinophils, and basophils in the BALF of rats with OVA-induced asthma. In addition, cordycepin with budesonide could reduce the cytokine expression levels of IL-5, IL-13, and TNF-*α* and the IgE level more than cordycepin without budesonide. The authors also found that cordycepin with budesonide more greatly reduced the thickness of the bronchial wall than cordycepin without budesonide. Notably, they observed that cordycepin with or without budesonide could markedly inhibit the p38-MAPK pathway and upregulate the adenosine A_2A_ receptor. However, budesonide alone did not have the same effect. The researchers revealed that the synergistic effects of cordycepin with budesonide on cytokine expression, IgE secretion, and airway remodeling might be due to the p38-MAPK pathway and A_2A_ receptor.

Cordycepin was discovered to regulate neuroimmune response by Du’s group (2019). They demonstrated that the administration of cordycepin through *IP* injection in mice model could reduce anxious behavior. The authors further evaluated the possible mechanism and found that cordycepin could increase IL-4 and IL-10 expression and decrease TNF-*α* and IL-1*β* expression in prefrontal cortex. IL-4 expression has now been demonstrated to be very strongly positively correlated with reduced anxiety (Gao et al., 2019).

Cordycepin was shown to suppress T cell activity by Yang’s group (2020) (Wang et al., 2020). Mice received IP injections of different concentrations of cordycepin daily for 2 weeks, followed by an intradermal injection of Complete Freund’s Adjuvant into their hind paw to test for an anti-inflammation effect. They found that cordycepin could reduce inflammation phenomenon including the thymus inhibition, spleen enlargement, and the reduction of edema and T cell infiltration in mice paws. In addition, they showed that cordycepin could reduce T cell proliferation and IL-2 expression. They further investigated the possible mechanism and found that cordycepin could inhibit the T cell receptor signaling pathway by reducing TAP-70 and Erk phosphorylation.

In this section, we found that cordycepin is capable of suppressing macrophage activity by, for example, reducing the expression of iNOS, COX-2, and TNF-*α*, which might relieve some disease symptoms—such as those of type II diabetes and traumatic brain injury. Researchers have also indicated that cordycepin can directly inhibit T cell activation and proliferation after stimulation through injection with Complete Freund'|’s Adjuvant, a widely accepted Th1-response dominant type of stimulation. Cordycepin can act as an anxiolytic reagent because it upregulates IL-4 expression in prefrontal cortex, which is negatively correlated with the frequency of anxious behavior. In addition, some researchers have indicated that cordycepin has some beneficial effects in OVA-induced asthma and DNFB-induced delayed-type hypersensitivity reaction.

### Trace Elements and Powders

Trace elements are essential to immune regulation and infection prevention (Li et al., 2019). The action of *C. militaris* trace elements and their ability to counteract the influenza virus were analyzed by Wang and Hou (2011) (Wang and Hou, 2011). They orally administered 4 mg/kg/day of *C. militaris* extracts to treat H1N1-infected mice and monitored mortality and levels of Cu, Zn, Mg, Mn, Se, and Fe in the serum. The researchers illustrated that H1N1-infected mice treated with the extract had lower mortality than did the nontreated controls (survival rates of all control and *C. militaris* treatment groups were 25 and 50%, respectively). The concentrations of Cu, Zn, Mg, Mn, Se, and Fe in the serum (μg/ml) were 1.11 ± 0.51, 0.69 ± 0.53, 15.68 ± 1.53, 0.008 ± 0.53, 0.26 ± 0.53, and 0.16 ± 0.03, respectively. Zn, Mg, and Se were significantly increased in mice treated with *C. militaris* extracts compared with corresponding levels in the controls. The researchers posited that Zn, Mg, and Se might play direct or indirect roles in counteracting the influenza virus through stimulating the host immune response.

The effect of *C. militaris* powder on the immunoregulation function of healthy volunteers was investigated by Sun et al. (Sun et al., 2014). The researchers treated 12 volunteers with 0.5, 1.5, or 3 g of *C. militaris* powder for one day and then analyzed and compared their blood samples before and after treatment. In the low-dosage group, the authors observed that eotaxin, fibroblast growth factor-2, growth regulated oncogene, and monocyte chemoattractant protein-1 were downregulated after treatment with the extract. In the medium-dosage group, the expression of growth regulated oncogene, sCD40L, and TNF-*α* were downregulated, and in the high-dosage group, IL-12, IFN-*γ*, inducible protein 10, monocyte chemoattractant protein-1, and macrophage inflammatory protein-1*β* were reduced. The researchers suggested that *C. militaris* powder, by virtue of its ability to reduce the excessive immune response, may play an inhibitory role in tumorigenesis and serve as an immunomodulator to treat some diet- and autoimmune-related diseases.

The available data are limited, therefore the immunoregulation trends of trace elements and powders of *C. militaris* remain unclear.

## Discussion

**FIGURE 4:**
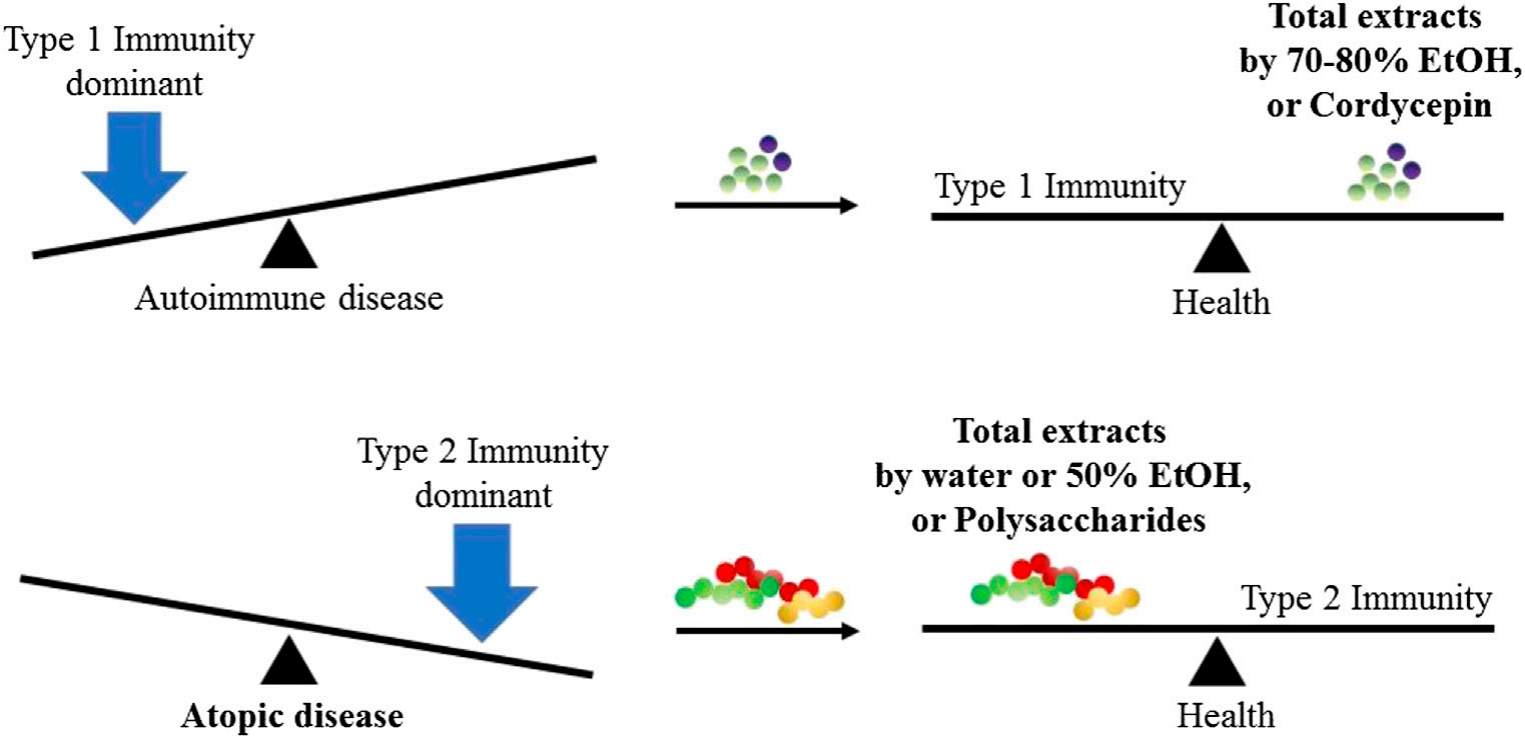
Immunomodulatory effects from different ingredients of *C. militaris*. In normal condition, host immune system always reach the balance of type 1 immunity and type 2 immunity. When the balance is broken, the host might suffer from the disease.

Novel researches on the immunomodulatory effects of *C. militaris* are summarized in Figure 4 according to its form: total extract, polysaccharides and cordycepin. Notably, we found that the investigators that used total extracts by water or 50% ethanol and the polysaccharides of *C. militaris* were likely to result in an immune response to type 1 immunity, such as increased TNF-*α* secretion in macrophages, decreased OVA-induced asthma in a mice model, or enhanced NK cells activities. In contrast, those using 70–80% ethanol extracts and cordycepin of *C. militaris* were more likely to obtain an immune response to type 2 immunity. However, some studies have indicated that cordycepin might also reduce OVA-induced asthma.

The immunomodulatory mechanism of total extract obtained using water or different concentration of ethanol is unclear. However, some studies have reported that the activation of macrophages and dendritic cells might be related with activating IL-18 or NF-*κ*B pathway (Kim et al., 2008; Shin, Kwon, et al., 2010; Shin, Park, et al., 2010) and the effect of antiallergic effects might be associated with lowering the expression level of the phosphorylation Syk, ERK, p38 and JNK (Oh et al., 2011). In addition, we suspect that the different immunomodulatory effects of *C. militaris* extracts obtained using different solvents may be associated with the polarity of the final product. Water extract with a low concentration of ethanol extract might produce high polarity product which prone to drive our immune system to type1 immunity; while a high concentration of ethanol extract might result in low polarity product that might drive our immune system to type2 immunity. The detail mechanisms of total extract by different solution should be further investigated.

The immunomodulatory mechanism of polysaccharide in *Dendrobium* (reported by the first author of this article in 2017) may be associated with TLR4. However, the polysaccharide in *C. militaris* appeared to be related not only to TLR4 but also to TLR2 and dectin-1 (Lee et al., 2017). In future research, we might compare the polysaccharide structures of these two commonly used herbs in traditional Chinese medicine to clarify their immunomodulatory effects. The immunomodulatory mechanism of cordycepin in different immune cells is related to different pathways. In macrophages, cordycepin reduces the activation of NF-*κ*B pathway; while in T-lymphocytes, cordycepin can suppress this activity and may be associated with the NFAT2 signaling pathway; finally, in mast cells, cordycepin can reduce the IL-13 and MDM2 expression in TSLP-induced HMC-1 cells.

The treatment of an excessive immune response caused by microbe infection, also known as a cytokine storm, is a critical field of research because it cytokine storms cause severe tissue damage, poor outcomes, and even death (Tisoncik et al., 2012; D'Elia et al., 2013; Guo and Thomas, 2017; Srikiatkhachorn et al., 2017). Here, we found that cordyceps extracts, namely a polysaccharide called APS, and trace elements from *C. militaris* might have beneficial effects in an influenza-induced cytokine storm and could reduce mortality in infected mice. Under normal conditions, a host immune system always reaches a balance between type 1 and type 2 immunity. When the balance is broken, the host might become ill. In this review, we found that total extracts using 70–80% ethyl alcohol and cordycepin of *C. militaris* were likely to result in type 2 host immunity, whereas polysaccharides and total extract by water or 50% ethyl alcohol of *C. militaris* were likely to result in type 1 immunity. This conclusion might serve as a principle to adjust our immune system and improve our health.

## Conclusion

We found that different *C. militaris* extracts have different immunomodulatory functions. Total extracts using water or 50% ethanol and polysaccharides from *C. militaris* could be used to treat tumors, allergies, and viral infections because these ingredients tend to drive the immune response toward type 1 immunity. Total extracts using 70–80% ethanol and cordycepin from *C. militaris* could be used as immunosuppressive agents for delayed-type hypersensitivity and tend to drive the immune response toward type 2 immunity. Thus, this review elucidated the bioactive compounds of *C. militaris* and their immunomodulatory effects.

## Author Contributions

CTL, KSH, JFS, JRC, WSK, GS, AMG, AHH and CHY organized the main text. CTL, KSH, YTW, JSW, YPH, YML, HHH, and CHY prepared the Figure and Tables.

## Funding

This work was financially supported by grants from I-Shou University and the Ministry of Science and Technology, Taiwan.

## Conflict of Interest

The authors declare that the research was conducted in the absence of any commercial or financial relationships that could be construed as a potential conflict of interest.

## Glossary

Ara: arabinose
BBB: blood-brain barrier
BALF: bronchoalveolar lavage fluid
JNK: c-Jun N-terminal kinase
CD: cluster of differentiation
*C. militaris*: *Cordyceps militaris*
cy: cyclophosphamide
COX-2: cycloxygenase-2
DNFB: 2,4-dinitro-1-fluorobenzene
ERK: extracellular-signal-regulated kinase
Gal: galactose
Glc: glucose
EC50: half maximal effective concentration
IC50: half maximal inhibitory concentration
Th: helper T
IgE: immunoglobulin E
iNOS: inducible nitric oxide synthase
IFN: interferon
IL: interleukin
ICAM-1: intracellular adhesion molecules-1
IP: intraperitoneal
LPS: lipopolysaccharides
IKKγ: IκB kinase gamma
MHC: major histocompatibility complex
Man: mannose
MAPK: mitogen-activated protein kinase
MW: molecular weight
MYD88: myeloid differentiation primary response 88
NK: natural killer
NEMO: NF-κB essential modifier
NFAT2: nuclear factor of activated T cells 2
NF-κB: nuclear factor kappa-light-chain-enhancer of activated B cells
IκBα: nuclear factor of kappa-light polypeptide gene enhancer in B-cells inhibitor, alpha
OVA: ovalbumin
p38: p38 mitogen-activated protein kinases
poly: ADP-ribose polymerase (PARP
PGE2: prostaglandin E2
ROS: reactive oxygen species
Rha: rhamnose
TSLP: thymic stromal lymphopoietin
TLR: toll-like receptor
TNF-α: tumor necrosis factor-α
Syk: tyrosine-protein kinase
Xyl: xylose

## References

[B1] Barcelos M. C. S. Vespermann K. A. C. Pelissari F. M. Molina G. (2020). Current status of biotechnological production and applications of microbial exopolysaccharides. Crit. Rev. Food Sci. Nutr. 60 (9), 1475–1495. 10.1080/10408398.2019.1575791. 30740985

[B2] Beltrame G. Trygg J. Rahkila J. Leino R. Yang B. (2019). Structural investigation of cell wall polysaccharides extracted from wild Finnish mushroom *Craterellus tubaeformis* (Funnel Chanterelle). Food Chem. 301, 125255 10.1016/j.foodchem.2019.125255. 31377617

[B3] Bi S. Huang W. Chen S. Huang C. Li C. Guo Z. 2020). Cordyceps militaris polysaccharide converts immunosuppressive macrophages into M1-like phenotype and activates T lymphocytes by inhibiting the PD-L1/PD-1 axis between TAMs and T lymphocytes. Int. J. Biol. Macromol. 150, 261–280. 10.1016/j.ijbiomac.2020.02.050. 32044366

[B4] Bieber T. (2008). Atopic dermatitis. N. Engl. J. Med. 358 (14), 1483–1494. 10.1056/nejmra074081. 18385500

[B5] Cheung J. K. H. Li J. Cheung A. W. H. Zhu Y. Zheng K. Y. Z. Bi C. W. C. 2009). Cordysinocan, a polysaccharide isolated from cultured Cordyceps, activates immune responses in cultured T-lymphocytes and macrophages: signaling cascade and induction of cytokines. J. Ethnopharmacol. 124 (1), 61–68. 10.1016/j.jep.2009.04.010. 19446414

[B6] Choi Y. H. Kim G.-Y. Lee H. H. (2014). Anti-inflammatory effects of cordycepin in lipopolysaccharide-stimulated RAW 264.7 macrophages through Toll-like receptor 4-mediated suppression of mitogen-activated protein kinases and NF-κB signaling pathways. Dddt 8, 1941–1953. 10.2147/dddt.s71957. 25342887 PMC4206205

[B7] Chu H.-L. Chien J.-C. Duh P.-D. (2011). Protective effect of *Cordyceps militaris* against high glucose-induced oxidative stress in human umbilical vein endothelial cells. Food Chem. 129 (3), 871–876. 10.1016/j.foodchem.2011.05.037. 25212312

[B8] Cui J. D. (2015). Biotechnological production and applications ofCordyceps militaris, a valued traditional Chinese medicine. Crit. Rev. Biotechnol. 35 (4), 475–484. 10.3109/07388551.2014.900604. 24666119

[B9] Cunningham K. G. Manson W. Spring F. S. Hutchinson S. A. (1950). Cordycepin, a metabolic product isolated from cultures of *Cordyceps militaris* (Linn.) Link. Nature 166 (4231), 949 10.1038/166949a0. 14796634

[B10] D'Elia R. V. Harrison K. Oyston P. C. Lukaszewski R. A. Clark G. C. (2013). Targeting the “cytokine storm” for therapeutic benefit. Clin. Vaccine Immunol. 20 (3), 319–327. 10.1128/cvi.00636-12. 23283640 PMC3592351

[B11] Das S. K. Masuda M. Sakurai A. Sakakibara M. (2010). Medicinal uses of the mushroom *Cordyceps militaris*: current state and prospects. Fitoterapia 81 (8), 961–968. 10.1016/j.fitote.2010.07.010. 20650308

[B12] Dong J. Z. Ding J. Yu P. Z. Lei C. Zheng X. J. Wang Y. (2013). Composition and distribution of the main active components in selenium-enriched fruit bodies of *Cordyceps militaris* link. Food Chem. 137 (1–4), 164–167. 10.1016/j.foodchem.2012.10.021. 23200005

[B13] Fei X. Zhang X. Zhang G.-q. Bao W.-p. Zhang Y.-y. Zhang M. (2017). Cordycepin inhibits airway remodeling in a rat model of chronic asthma. Biomed. Pharmacother. 88, 335–341. 10.1016/j.biopha.2017.01.025. 28119235

[B14] Fitzgerald K. A. Kagan J. C. (2020). Toll-like receptors and the control of immunity. Cell 180 (6), 1044–1066. 10.1016/j.cell.2020.02.041. 32164908 PMC9358771

[B15] Gao T. Li B. Hou Y. Luo S. Feng L. Nie J. (2019). Interleukin-4 signalling pathway underlies the anxiolytic effect induced by 3-deoxyadenosine. Psychopharmacology 236 (10), 2959–2973. 10.1007/s00213-019-5186-7. 30963194

[B16] Gause W. C. Wynn T. A. Allen J. E. (2013). Type 2 immunity and wound healing: evolutionary refinement of adaptive immunity by helminths. Nat. Rev. Immunol. 13 (8), 607–614. 10.1038/nri3476. 23827958 PMC3789590

[B17] Gieseck R. L. III. Wilson M. S. Wynn T. A. (2018). Type 2 immunity in tissue repair and fibrosis. Nat. Rev. Immunol. 18 (1), 62–76. 10.1038/nri.2017.90. 28853443

[B18] Guo X.-z. J. Thomas P. G. (2017). New fronts emerge in the influenza cytokine storm. Semin. Immunopathol. 39 (5), 541–550. 10.1007/s00281-017-0636-y. 28555383 PMC5580809

[B19] Guo Z. Chen W. Dai G. Huang Y. (2020). Cordycepin suppresses the migration and invasion of human liver cancer cells by downregulating the expression of CXCR4. Int. J. Mol. Med. 45 (1), 141–150. 10.3892/ijmm.2019.4391. 31746344 PMC6889938

[B20] Han E. S. Oh J. Y. Park H.-J. (2011). *Cordyceps militaris* extract suppresses dextran sodium sulfate-induced acute colitis in mice and production of inflammatory mediators from macrophages and mast cells. J. Ethnopharmacol. 134 (3), 703–710. 10.1016/j.jep.2011.01.022. 21277968

[B21] He B.-L. Zheng Q.-W. Guo L.-Q. Huang J.-Y. Yun F. Huang S.-S. (2020). Structural characterization and immune-enhancing activity of a novel high-molecular-weight polysaccharide from *Cordyceps militaris* . Int. J. Biol. Macromol. 145, 11–20. 10.1016/j.ijbiomac.2019.12.115. 31846656

[B22] He X. Fang J. Guo Q. Wang M. Li Y. Meng Y. (2020). Advances in antiviral polysaccharides derived from edible and medicinal plants and mushrooms. Carbohydr. Polym. 229, 115548 10.1016/j.carbpol.2019.115548. 31826474

[B23] Hsu C.-H. Sun H.-L. Sheu J.-N. Ku M.-S. Hu C.-M. Chan Y. (2008). Effects of the immunomodulatory agent *Cordyceps militaris* on airway inflammation in a mouse asthma model. Pediatrics & Neonatology 49 (5), 171–178. 10.1016/s1875-9572(09)60004-8. 19133568

[B24] Jeong J.-W. Jin C.-Y. Kim G.-Y. Lee J.-D. Park C. Kim G.-D. (2010). Anti-inflammatory effects of cordycepin via suppression of inflammatory mediators in BV2 microglial cells. Int. Immunopharm. 10 (12), 1580–1586. 10.1016/j.intimp.2010.09.011. 20937401

[B25] Kang H. J. Baik H. W. Kim S. J. Lee S. G. Ahn H. Y. Park J. S. (2015). Cordyceps militarisEnhances cell-mediated immunity in healthy Korean men. J. Med. Food 18 (10), 1164–1172. 10.1089/jmf.2014.3350. 26284906

[B26] Kawai T. Akira S. (2010). The role of pattern-recognition receptors in innate immunity: update on toll-like receptors. Nat. Immunol. 11 (5), 373–384. 10.1038/ni.1863. 20404851

[B27] Kay A. B. (2001a). Allergy and allergic diseases. N. Engl. J. Med. 344 (1), 30–37. 10.1056/nejm200101043440106. 11136958

[B28] Kay A. B. (2001b). Allergy and allergic diseases. N. Engl. J. Med. 344 (2), 109–113. 10.1056/nejm200101113440206. 11150362

[B29] Khan M. A. Tania M. (2020). Cordycepin in anticancer research: molecular mechanism of therapeutic effects. Cmc 27 (6), 983–996. 10.2174/0929867325666181001105749. 30277143

[B30] Kim C. S. Lee S.-Y. Cho S.-H. Ko Y.-M. Kim B.-H. Kim H.-J. (2008). Cordyceps militaris induces the IL-18 expression via its promoter activation for IFN-γ production. J. Ethnopharmacol. 120 (3), 366–371. 10.1016/j.jep.2008.09.010. 18929637

[B31] Kim H. G. Shrestha B. Lim S. Y. Yoon D. H. Chang W. C. Shin D. J. (2006). Cordycepin inhibits lipopolysaccharide-induced inflammation by the suppression of NF-kappaB through Akt and p38 inhibition in RAW 264.7 macrophage cells. Eur. J. Pharmacol. 545 (2–3), 192–199. 10.1016/j.ejphar.2006.06.047. 16899239

[B32] Kim H. S. Kim J. Y. Kang J. S. Kim H. M. Kim Y. O. Hong I. P. (2010). Cordlan polysaccharide isolated from mushroom *Cordyceps militaris* induces dendritic cell maturation through toll-like receptor 4 signalings. Food Chem. Toxicol. 48 (7), 1926–1933. 10.1016/j.fct.2010.04.036. 20434503

[B33] Kitamura M. Kato H. Saito Y. Nakajima S. Takahashi S. Johno H. (2011). Aberrant, differential and bidirectional regulation of the unfolded protein response towards cell survival by 3′-deoxyadenosine. Cell Death Differ. 18 (12), 1876–1888. 10.1038/cdd.2011.63. 21597460 PMC3214911

[B34] Lee C. T. Kuo H. C. Chen Y. H. Tsai M. Y. (2017). Current advances in the biological activity of polysaccharides in dendrobium with intriguing therapeutic potential. Curr. Med. Chem. 25 (14), 1663–1681. 10.2174/0929867324666170227114648 28245766

[B35] Lee H. H. Park H. Sung G.-H. Lee K. Lee T. Lee I. (2014). Anti-influenza effect of *Cordyceps militaris* through immunomodulation in a DBA/2 mouse model. J. Microbiol. 52 (8), 696–701. 10.1007/s12275-014-4300-0. 25037880

[B36] Lee J. S. Hong E. K. (2011). Immunostimulating activity of the polysaccharides isolated from *Cordyceps militaris* . Int. Immunopharm. 11 (9), 1226–1233. 10.1016/j.intimp.2011.04.001. 21497206

[B37] Lee J. S. Kwon D. S. Lee K. R. Park J. M. Ha S.-J. Hong E. K. (2015). Mechanism of macrophage activation induced by polysaccharide from *Cordyceps militaris* culture broth. Carbohydr. Polym. 120, 29–37. 10.1016/j.carbpol.2014.11.059. 25662684

[B38] Lee J. S. Kwon J. S. Won D. P. Lee K. E. Shin W. C. Hong E. K. (2010). Study on macrophage activation and structural characteristics of purified polysaccharide from the liquid culture broth of *Cordyceps militaris* . Carbohydr. Polym. 82, 982–988. 10.1016/j.carbpol.2010.06.025.

[B39] Lee J. S. Kwon J. S. Yun J. S. Pahk J. W. Shin W. C. Lee S. Y. (2010). Structural characterization of immunostimulating polysaccharide from cultured mycelia of *Cordyceps militaris* . Carbohydr. Polym. 80, 1011–1017. 10.1016/j.carbpol.2010.01.017.

[B40] Li J. Shen B. Nie S. Duan Z. Chen K. (2019). A combination of selenium and polysaccharides: promising therapeutic potential. Carbohydr. Polym. 206, 163–173. 10.1016/j.carbpol.2018.10.088. 30553309

[B41] Lin B. Li S. (2011). “Cordyceps as an herbal drug,” in Herbal medicine: biomolecular and clinical aspects. Editors Benzie I. F. F. Wachtel-Galor S. (Boca Raton, FL). 22593937

[B42] Lin Y.-S. Yang C.-H. Lu K. Huang K.-S. Zheng Y.-Z. (2011). Synthesis of agar microparticles using temperature-controlled microfluidic devices for *Cordyceps militaris* cultivation. Electrophoresis 32 (22), 3157–3163. 10.1002/elps.201100343. 22012813

[B43] Liu J.-y. Feng C.-p. Li X. Chang M.-c. Meng J.-l. Xu L.-j. (2016a). Immunomodulatory and antioxidative activity of *Cordyceps militaris* polysaccharides in mice. Int. J. Biol. Macromol. 86, 594–598. 10.1016/j.ijbiomac.2016.02.009. 26853825

[B44] Liu X.-C. Zhu Z.-Y. Tang Y.-L. Wang M.-f. Wang Z. Liu A.-J. (2016b). Structural properties of polysaccharides from cultivated fruit bodies and mycelium of *Cordyceps militaris* . Carbohydr. Polym. 142, 63–72. 10.1016/j.carbpol.2016.01.040. 26917375

[B45] Lloyd C. M. Snelgrove R. J. (2018). Type 2 immunity: expanding our view. Sci. Immunol. 3 (25), eaat1604 10.1126/sciimmunol.aat1604. 29980619

[B46] Lozupone C. A. (2018). Unraveling interactions between the microbiome and the host immune system to decipher mechanisms of disease. mSystems 3 (2), e00183–00117. 10.1128/msystems.00183-17. 29556546 PMC5853183

[B47] Lu R.-L. Bao G.-H. Hu F.-L. Huang B. Li C.-R. Li Z.-Z. (2014). Comparison of cytotoxic extracts from fruiting bodies, infected insects and cultured mycelia of *Cordyceps formosana* . Food Chem. 145, 1066–1071. 10.1016/j.foodchem.2013.09.001. 24128585

[B48] Luo X. Duan Y. Yang W. Zhang H. Li C. Zhang J. (2017). Structural elucidation and immunostimulatory activity of polysaccharide isolated by subcritical water extraction from *Cordyceps militaris* . Carbohydr. Polym. 157, 794–802. 10.1016/j.carbpol.2016.10.066. 27987993

[B49] McInnes I. B. Schett G. (2011). The pathogenesis of rheumatoid arthritis. N. Engl. J. Med. 365 (23), 2205–2219. 10.1056/nejmra1004965. 22150039

[B50] Meng X. Li Y. Li S. Gan R.-Y. Li H.-B. (2018). Natural products for prevention and treatment of chemical-induced liver injuries. Compr. Rev. Food Sci. Food Saf. 17 (2), 472–495. 10.1111/1541-4337.12335. 33350084

[B51] Mohan K. Muralisankar T. Uthayakumar V. Chandirasekar R. Revathi N. Ramu Ganesan A. 2020). Trends in the extraction, purification, characterisation and biological activities of polysaccharides from tropical and sub-tropical fruits - a comprehensive review. Carbohydr. Polym. 238, 116185 10.1016/j.carbpol.2020.116185. 32299552

[B52] Moutsopoulos N. M. Zerbe C. S. Wild T. Dutzan N. Brenchley L. DiPasquale G. 2017). Interleukin-12 and interleukin-23 blockade in leukocyte adhesion deficiency type 1. N. Engl. J. Med. 376 (12), 1141–1146. 10.1056/nejmoa1612197. 28328326 PMC5494261

[B100] Ng T. B., Wang H. X. (2005). Pharmacological actions of Cordyceps, a prized folk medicine. J Pharm Pharmacol., 57 (12), 1509–1519. 10.1211/jpp.57.12.0001. 16354395

[B53] Oh J. Y. Choi W.-S. Lee C. H. Park H.-J. (2011). The ethyl acetate extract of *Cordyceps militaris* inhibits IgE-mediated allergic responses in mast cells and passive cutaneous anaphylaxis reaction in mice. J. Ethnopharmacol. 135 (2), 422–429. 10.1016/j.jep.2011.03.030. 21420483

[B54] Ohta Y. Lee J.-B. Hayashi K. Fujita A. Park D. K. Hayashi T. (2007). *In vivo* anti-influenza virus activity of an immunomodulatory acidic polysaccharide isolated from *Cordyceps militaris* grown on germinated soybeans. J. Agric. Food Chem. 55 (25), 10194–10199. 10.1021/jf0721287. 17988090

[B55] Park H.-J. (2015). Ethanol extract of *Cordyceps militaris* grown on germinated soybeans inhibits 2, 4-dinitrophenolfluorobenzene-induced allergic contact dermatitis. Journal of Functional Foods 17, 938–947. 10.1016/j.jff.2015.06.046.

[B56] Qin P. Li X. Yang H. Wang Z.-Y. Lu D. (2019). Therapeutic potential and biological applications of Cordycepin and metabolic mechanisms in Cordycepin-producing fungi. Molecules 24 (12), 2231 10.3390/molecules24122231. PMC6632035 31207985

[B57] Ren Z. Cui J. Huo Z. Xue J. Cui H. Luo B. (2012). Cordycepin suppresses TNF-α-induced NF-κB activation by reducing p65 transcriptional activity, inhibiting IκBα phosphorylation, and blocking IKKγ ubiquitination. Int. Immunopharm. 14 (4), 698–703. 10.1016/j.intimp.2012.10.008. 23102662

[B58] Rodrigues C. Sousa C. Lopes J. A. Novais Â. Peixe L. (2020). A front line on klebsiella pneumoniae capsular polysaccharide knowledge: fourier transform infrared spectroscopy as an accurate and fast typing tool. mSystems 5 (2), e00386–00319. 10.1128/msystems.00386-19. 32209717 PMC7093823

[B59] Ruthes A. C. Smiderle F. R. Iacomini M. (2016). Mushroom heteropolysaccharides: a review on their sources, structure and biological effects. Carbohydr. Polym. 136, 358–375. 10.1016/j.carbpol.2015.08.061. 26572366

[B60] Sacks D. Sacks D. Baxter B. Campbell B. C. V. Carpenter J. S. Cognard C. 2018). Multisociety consensus quality improvement revised consensus statement for endovascular therapy of acute ischemic stroke. Int. J. Stroke 13 (6), 612–632. 10.1177/1747493018778713. 29786478

[B61] Schnaar R. L. (2016). Glycobiology simplified: diverse roles of glycan recognition in inflammation. J. Leukoc. Biol. 99 (6), 825–838. 10.1189/jlb.3ri0116-021r. 27004978 PMC4952015

[B62] Shapouri-Moghaddam A. Mohammadian S. Vazini H. Taghadosi M. Esmaeili S.-A. Mardani F. (2018). Macrophage plasticity, polarization, and function in health and disease. J. Cell. Physiol. 233 (9), 6425–6440. 10.1002/jcp.26429. PMC13482560 29319160

[B63] Shen H.-S. Shao S. Chen J.-C. Zhou T. (2017). Antimicrobials from mushrooms for assuring food safety. Compr. Rev. Food Sci. Food Saf. 16 (2), 316–329. 10.1111/1541-4337.12255. 33371536

[B64] Shin S. Kwon J. Lee S. Kong H. Lee S. Lee C.-K. (2010). Immunostimulatory effects ofCordyceps militarison macrophages through the enhanced production of cytokines via the activation of NF-κB. Immune Netw 10 (2), 55–63. 10.4110/in.2010.10.2.55. 20532125 PMC2881426

[B65] Shin S. Lee S. Kwon J. Moon S. Lee S. Lee C.-K. (2009). Cordycepin suppresses expression of diabetes regulating genes by inhibition of lipopolysaccharide-induced inflammation in macrophages. Immune Netw 9 (3), 98–105. 10.4110/in.2009.9.3.98. 20107539 PMC2803303

[B66] Shin S. Moon S. Park Y. Kwon J. Lee S. Lee C.-K. (2009). Role of cordycepin and adenosine on the phenotypic switch of macrophages via induced anti-inflammatory cytokines. Immune Netw 9 (6), 255–264. 10.4110/in.2009.9.6.255. 20157613 PMC2816959

[B67] Shin S. Park Y. Kim S. Oh H.-E. Ko Y.-W. Han S. (2010). Cordyceps militarisEnhances MHC-restricted antigen presentation via the induced expression of MHC molecules and production of cytokines. Immune Netw 10 (4), 135–143. 10.4110/in.2010.10.4.135. 20844738 PMC2939358

[B68] Sims I. M. Frese S. A. Walter J. Loach D. Wilson M. Appleyard K. 2011). Structure and functions of exopolysaccharide produced by gut commensal *Lactobacillus reuteri* 100-23. ISME J. 5 (7), 1115–1124. 10.1038/ismej.2010.201. 21248858 PMC3146279

[B69] Srikiatkhachorn A. Mathew A. Rothman A. L. (2017). Immune-mediated cytokine storm and its role in severe dengue. Semin. Immunopathol. 39 (5), 563–574. 10.1007/s00281-017-0625-1. 28401256 PMC5496927

[B70] Sun T. Dong W. Jiang G. Yang J. Liu J. Zhao L. (2019). *Cordyceps militaris* improves chronic kidney disease by affecting TLR4/NF-kappaB redox signaling pathway. Oxidative Medicine and Cellular Longevity 2019, 7850863 10.1155/2019/7850863. 31049139 PMC6462325

[B71] Sun Y. Shao Y. Zhang Z. Wang L. Mariga A. M. Pang G. (2014). Regulation of human cytokines by *Cordyceps militaris* . J. Food Drug Anal. 22 (4), 463–467. 10.1016/j.jfda.2014.01.025. 28911461 PMC9354998

[B72] Sun Y. Zhang M. Fang Z. (2019). Efficient physical extraction of active constituents from edible fungi and their potential bioactivities: a review. Trends in food science and technology 105, 468–482. 10.1016/j.tifs.2019.02.02

[B73] Tisoncik J. R. Korth M. J. Simmons C. P. Farrar J. Martin T. R. Katze M. G. (2012). Into the eye of the cytokine storm. Microbiol. Mol. Biol. Rev. 76 (1), 16–32. 10.1128/mmbr.05015-11. 22390970 PMC3294426

[B74] Wang C. Wang S. (2017). Insect pathogenic fungi: genomics, molecular interactions, and genetic improvements. Annu. Rev. Entomol. 62, 73–90. 10.1146/annurev-ento-031616-035509. 27860524

[B75] Wang L. Hou Y. (2011). Determination of trace elements in anti-influenza virus mushrooms. Biol. Trace Elem. Res. 143 (3), 1799–1807. 10.1007/s12011-011-8986-0. 21301988

[B76] Wang M. Meng X. Y. Yang R. L. Qin T. Wang X. Y. Zhang K. Y. 2012). *Cordyceps militaris* polysaccharides can enhance the immunity and antioxidation activity in immunosuppressed mice. Carbohydr. Polym. 89 (2), 461–466. 10.1016/j.carbpol.2012.03.029. 24750744

[B77] Wang X. Xi D. Mo J. Wang K. Luo Y. Xia E. (2020). Cordycepin exhibits a suppressive effect on T cells through inhibiting TCR signaling cascade in CFA-induced inflammation mice model. Immunopharmacol. Immunotoxicol. 42 (2), 119–127. 10.1080/08923973.2020.1728310. 32105161

[B78] Wang Y. Stata M. Wang W. Stajich J. E. White M. M. Moncalvo J.-M. (2018). Comparative genomics reveals the core gene toolbox for the fungus-insect symbiosis. mBio 9 (3), e00636–18. 10.1128/mbio.00636-18. 29764946 PMC5954228

[B79] Wang Z. Chen Z. Jiang Z. Luo P. Liu L. Huang Y. (2019). Cordycepin prevents radiation ulcer by inhibiting cell senescence via NRF2 and AMPK in rodents. Nat. Commun. 10 (1), 2538 10.1038/s41467-019-10386-8. 31182708 PMC6557849

[B80] Won S.-Y. Park E.-H. (2005). Anti-inflammatory and related pharmacological activities of cultured mycelia and fruiting bodies of *Cordyceps militaris* . J. Ethnopharmacol. 96 (3), 555–561. 10.1016/j.jep.2004.10.009. 15619578

[B81] Wu L. Sun H. Hao Y. Zheng X. Song Q. Dai S. (2020). Chemical structure and inhibition on α-glucosidase of the polysaccharides from Cordyceps militaris with different developmental stages. Int. J. Biol. Macromol. 148, 722–736. 10.1016/j.ijbiomac.2020.01.178. 31972201

[B82] Xiang R. K. Zhen Y. Z. Xiao J. Z. Yong M. Z. (2020). Effects of cordyceps polysaccharides on pasting properties and *in vitro* starch digestibility of wheat starch. Food Hydrocolloids 102, 1–8. 10.1016/j.foodhyd.2019.105604

[B83] Xiong Y. Zhang S. Xu L. Song B. Huang G. Lu J. (2013). Suppression of T-cell activation *in vitro* and *in vivo* by cordycepin from *Cordyceps militaris* . J. Surg. Res. 185 (2), 912–922. 10.1016/j.jss.2013.06.057. 23927879

[B84] Yamaguchi T. Takizawa F. Fischer U. Dijkstra J. (2015). Along the axis between Type 1 and Type 2 immunity; principles conserved in evolution from fish to mammals. Biology 4 (4), 814–859. 10.3390/biology4040814. 26593954 PMC4690019

[B85] Yang C.-H. Kao Y.-H. Huang K.-S. Wang C.-Y. Lin L.-W. (2012). *Cordyceps militaris* and mycelial fermentation induced apoptosis and autophagy of human glioblastoma cells. Cell Death Dis. 3, e431 10.1038/cddis.2012.172. 23190603 PMC3542607

[B86] Yang M. Belwal T. Devkota H. P. Li L. Luo Z. (2019). Trends of utilizing mushroom polysaccharides (MPs) as potent nutraceutical components in food and medicine: a comprehensive review. Trends Food Sci. Technol. 92, 94–110. 10.1016/j.tifs.2019.08.009

[B87] Yang X. Li Y. He Y. Li T. Wang W. Zhang J. (2015). Cordycepin alleviates airway hyperreactivity in a murine model of asthma by attenuating the inflammatory process. Int. Immunopharm. 26 (2), 401–408. 10.1016/j.intimp.2015.04.017. 25912153

[B88] Yoon S. Park S. Park Y. (2018). The anticancer properties of cordycepin and their underlying mechanisms. Ijms 19 (10), 3027 10.3390/ijms19103027. PMC6212910 30287757

[B89] Yoou M.-s. Yoon K. W. Choi Y. Kim H.-M. Jeong H.-J. (2017). Cordycepin diminishes thymic stromal lymphopoietin-induced interleukin-13 production. Eur. J. Pharmacol. 802, 1–6. 10.1016/j.ejphar.2017.02.033. 28219709

[B90] Yu R. Song L. Zhao Y. Bin W. Wang L. Zhang H. (2004). Isolation and biological properties of polysaccharide CPS-1 from cultured *Cordyceps militaris* . Fitoterapia 75 (5), 465–472. 10.1016/j.fitote.2004.04.003. 15261384

[B91] Yuan J. Wang A. He Y. Si Z. Xu S. Zhang S. (2016). Cordycepin attenuates traumatic brain injury-induced impairments of blood-brain barrier integrity in rats. Brain Res. Bull. 127, 171–176. 10.1016/j.brainresbull.2016.09.010. 27646481

[B92] Yue K. Ye M. Zhou Z. Sun W. Lin X. (2013). The genusCordyceps: a chemical and pharmacological review. J. Pharm. Pharmacol. 65 (4), 474–493. 10.1111/j.2042-7158.2012.01601.x. 23488776

[B93] Zhang A.-X. Mouhoumed A.-Z. Tong S.-M. Ying S.-H. Feng M.-G. (2019). BrlA and AbaA govern virulence-required dimorphic switch, conidiation, and pathogenicity in a fungal insect pathogen. mSystems 4 (4), e00140 10.1128/msystems.00140-19. 31289140 PMC6616149

[B94] Zhang F. L. Yang X. F. Wang D. Lei S. R. Guo L. A. Liu W. J. (2020a). A simple and effective method to discern the true commercial Chinese cordyceps from counterfeits. Sci. Rep. 10 (1), 2974 10.1038/s41598-020-59900-9. 32076084 PMC7031310

[B95] Zhang J. Wen C. Duan Y. Zhang H. Ma H. (2019). Advance in *Cordyceps militaris* (Linn) link polysaccharides: isolation, structure, and bioactivities: a review. Int. J. Biol. Macromol. 132, 906–914. 10.1016/j.ijbiomac.2019.04.020. 30954592

[B96] Zhang J. Wen C. Zhang H. Duan Y. Ma H. (2020b). Recent advances in the extraction of bioactive compounds with subcritical water: a review. Trends Food Sci. Technol. 95, 183–195. 10.1016/j.tifs.2019.11.018.

[B97] Zhang Y. Zeng Y. Cui Y. Liu H. Dong C. Sun Y. (2020c). Structural characterization, antioxidant and immunomodulatory activities of a neutral polysaccharide from *Cordyceps militaris* cultivated on hull-less barley. Carbohydr. Polym. 235, 115969 10.1016/j.carbpol.2020.115969. 32122503

[B98] Zheng L.-Y. Guo X.-S. He B. Sun L.-J. Peng Y. Dong S.-S. (2011). Genome-wide patterns of genetic variation in sweet and grain sorghum (Sorghum bicolor). Genome Biol. 12 (11), R114 10.1186/gb-2011-12-11-r114. 22104744 PMC3334600

[B99] Zhu S.-j. Pan J. Zhao B. Liang J. Ze-Yu W. Yang J.-j. (2013). Comparisons on enhancing the immunity of fresh and dry *Cordyceps militaris in vivo* and *in vitro* . J. Ethnopharmacol. 149 (3), 713–719. 10.1016/j.jep.2013.07.037. 23916792

